# Multiscale analysis of pangenomes enables improved representation of genomic diversity for repetitive and clinically relevant genes

**DOI:** 10.1038/s41592-023-01914-y

**Published:** 2023-06-26

**Authors:** Chen-Shan Chin, Sairam Behera, Asif Khalak, Fritz J. Sedlazeck, Peter H. Sudmant, Justin Wagner, Justin M. Zook

**Affiliations:** 1 GeneDX, Stamford, CT USA; 2 Foundation of Biological Data Science, Belmont, CA USA; 3 Human Genome Sequencing Center, Baylor College of Medicine, Houston, TX USA; 4 Department of Computer Science, Rice University, Houston, TX USA; 5 Department of Integrative Biology, University of California Berkeley, Berkeley, CA USA; 6 Material Measurement Laboratory, National Institute of Standards and Technology, Gaithersburg, MD USA

**Keywords:** Software, Genome assembly algorithms, Structural variation, Genome assembly algorithms

## Abstract

Advancements in sequencing technologies and assembly methods enable the regular production of high-quality genome assemblies characterizing complex regions. However, challenges remain in efficiently interpreting variation at various scales, from smaller tandem repeats to megabase rearrangements, across many human genomes. We present a PanGenome Research Tool Kit (PGR-TK) enabling analyses of complex pangenome structural and haplotype variation at multiple scales. We apply the graph decomposition methods in PGR-TK to the class II major histocompatibility complex demonstrating the importance of the human pangenome for analyzing complicated regions. Moreover, we investigate the Y-chromosome genes, *DAZ1*/*DAZ2*/*DAZ3*/*DAZ4*, of which structural variants have been linked to male infertility, and X-chromosome genes *OPN1LW* and *OPN1MW* linked to eye disorders. We further showcase PGR-TK across 395 complex repetitive medically important genes. This highlights the power of PGR-TK to resolve complex variation in regions of the genome that were previously too complex to analyze.

## Abstract (web summary)

The PanGenome Research Tool Kit (PGR-TK) achieves flexible and scalable representation, visualization and analysis of genomic variation using pangenome graphs.

## Main

Studying genomes, the fundamental information contained in all living beings, is the foundation for understanding the biology and evolution of all organisms, as well as the genetic diseases of humans. Despite the millions of human genomes that have been sequenced since the onset of the Human Genome Project^1,2^ and the dramatic reduction in the cost of short-read (roughly 150 bp) DNA sequencing, there is still fundamental information yet to be revealed in genomics^3^. While it is important to recognize successes so far, including small variant surveys, genome-wide association studies^4–7^ and the development of routine laboratory tests for genetic-based precision medicine^8–11^, there remain fundamental biological questions that involve structures at greater length scales that can only be captured using long-range information accessible by long-read technologies and diploid phased assemblies^12–14^.

With the possibility of resolving variants at multiple scales, small and large, researchers now can fully characterize previously inaccessible regions by focusing on single-nucleotide polymorphisms and small indels alone^15,16^. Examples of such previously inaccessible regions include centromere, telomeres and complex repeat regions. Recent results with pangenome-scale de novo human assemblies and the CHM13 telomere to telomere assembly have already shown the potential for revealing biological insights^3,17–20^, which are the foundation for understanding complex genetic diseases.

A concept that becomes powerful in such analyses is that of the pangenome: that is, a characterization of both the genetic structure and the genetic variation across diverse individuals of a species. However, such complexity and diversity generate interpretive challenges that require more advanced tools. A graph representing many genome assemblies at once provides a way to visualize and analyze complicated structural variations among different haplotypes^21–28^. Previously, distinct approaches to generate graphs representing pangenome structures have been proposed for various applications. For example, variant graph^27,29^ and PanGenie^26^ focus on improving variant calling and genotyping with pangenome references. Cactus graphs^25^, Progressive Cactus graphs^30^, PGGB^19^ and cactus-mingraph^19,24^ build pangenome graphs aiming for large-scale structural rearrangement comparisons. Stringomics graph with ‘stringlet‘^21^, Seqwish^22^ and de Bruijn graph based approaches^26,31^ provide algorithms and data structures for improving storage and query efficiency and reduce bias caused by the alignment processes. These tools provide more accessible pictures for researchers to understand repeats and rearrangements than using computational intensive and visually complicated multiple sequence alignments (MSA)^30,32,33^. The traditional MSA view is typically represented as a big matrix where each row represents a different genome, and the columns represent the bases. With MSA, the relationships between sequences are not obvious when there are complicated repeats or structural variations. Instead of per base alignment, a pangenome graph effectively condenses the homologous regions and can express the relationship between those different regions through graph edge connections that are easier to trace. Meanwhile, although a graph is an elegant data structure for gathering information from pangenomic assemblies, there remains a gap in projecting the underlying linear sequences onto a graph at various scales to reveal and compare features of many different haplotypes^23^.

To address this gap, we present a generalized graph framework as a software package, PanGenome Research Tool Kit (PGR-TK, https://github.com/GeneDx/pgr-tk), that is scalable to rapidly represent multiple samples at varying resolution levels by adopting different parameters to facilitate exploratory analysis. PGR-TK is able to resolve and visualize the most complex regions of the human genome that often affect multiple medical important phenotypes (for example, *LPA*, *HLA* and so on).

We demonstrate the ability of PGR-TK to visualize and enable deeper insights into complex variants in repetitive genes, including a gene within nested palindromic and tandem repeats (*AMY1A*), the major histocompatibility complex (MHC) region including the complex human leukocyte antigen (HLA) class II locus^34^, the Genome in a Bottle (GIAB) challenging medically relevant gene list^35^ and chrX and chrY ampliconic genes^36^. Many genome-wide studies including GIAB have excluded many of these genes from their analyses because they are difficult to represent in VCF and it is challenging to compare differing representations^37^. To understand how PGR-TK can help with the challenge of variant calling, variant representation and comparison across these genes and genomic loci we use the Human Pangenome Reference Consortium (HPRC)^19,38^ year one 47 human genome assemblies (94 diverse haplotypes). With the ability to survey a large set of genes swiftly with PGR-TK, we hope to understand how to better provide a broader benchmark set for challenging genes using HPRC assemblies in the future. We examine *OPN1LW* and *OPN1MW* on chromosome X and *DAZ1*/*2*/*3*/*4* on chromosome Y in detail to understand how the limit due to complicated large-scale genome rearrangement affects the current methodology of generating variant call benchmarks. Our initial analysis of the GIAB clinical and medically important genes (CMRG) with a pangenome graph approach will help the research community to adapt the pangenome resource for clinical and medical genetic applications. Tools for visualizing and analyzing complicated rearrangement loci such as PGR-TK will be essential for better variant calling and understanding how structural variations with repeats affect the results for the community.

## Results

### PGR-TK

**Fig. 1:**
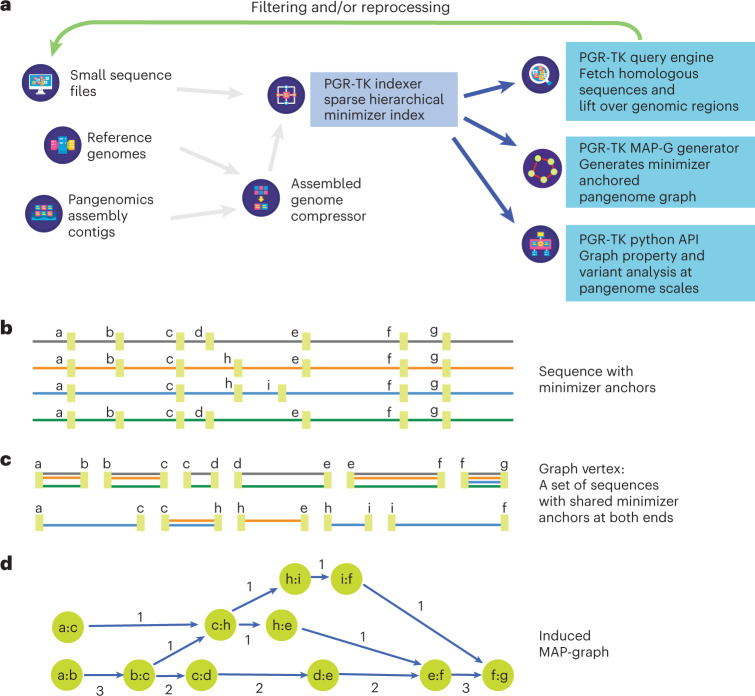
The architecture of PGR-TK and minimizer anchored graph construction. **a**, Overall architecture and design scope of the PGR-TK library. **b**, Each sequence in the database is scanned, and the location of the minimizers are recorded to construct the SHIMMER database and MAP-graph. **c**, Each vertex in the MAP-graph represents a collection of sequence fragments sharing the two ending SHIMMERs in the database. **d**, The MAP-graph is constructed by merging all paths from all sequences into a graph.

The PGR-TK has several different components to facilitate rapid pangenome analysis. The general scope and design of the PGR-TK is illustrated in Fig. 1a. PGR-TK applies the computation techniques and data structures initially developed for fast genome assemblers^39–41^ to pangenome analysis tasks. Instead of building a whole genome graph at once, which can be computationally expensive. PGR-TK provides tools for building an indexed sequence database, fetching and querying sequences of interest (for example, genes or regions with large-scale structural variations) from the database to create pangenomics graphs accordingly. It uses minimizer anchors to generate pangenome graphs at different scales without more computationally intensive sequence-to-sequence alignment or explicitly calling variants with respect to a reference. The generation step of the pangenome graph considers all input sequences equivalently without a preferential reference. Note that the sequence fetching step using a query sequence may introduce bias due to missing or incorrect alignments. We also developed an algorithm to decompose tangled pangenome graphs to more manageable units (principal bundles). With such decomposition, we can easily project the linear genomics sequence onto the principal bundles. It can provide more straightforward visualization to generate insight by revealing the contrast of the repeat and rearrangement variations among the haplotypes. Such pangenome-level graph decomposition provides utilities similar to the A-de Bruijn graph approach for identifying repeats and conserved segmental duplications^42–45^, but for the whole human pangenome collection at once.

PGR-TK uses the Assembly Genome Compressor^46^ for storing pangenome assembly contigs and includes binary for creating the sparse hierarchical minimizer (SHIMMER) index. For the HPRC year one data release (94 fully assembled haplotypes from 47 samples), it takes 18 minutes to create the index file on an Amazon Web Services c5.12xlarge instance, with the default parameters. This is substantially faster than building an alignment index for query with tools such as mimimap2 (Supplementary Table 1). Although PGR-TK was designed to retrieve homologous sequences from the database, rather than finding the best alignments, our evaluations indicate that the query results are generally consistent with other alignment tools (Supplementary Tables 2 and 3).

Once the index is built, it can be loaded into memory within minutes. As shown in Fig. 1a, there are three main functional modules using the index: (1) fetching homologous regions and sequences of the pangenome database given a query sequence, (2) creating a minimizer anchored pangenome-graph (MAP-graph) and (3) command line tools and a software library for interactive analysis and visualization on the generated graph and the underlying sequences. One of the main applications of PGR-TK is for deconvolving large regions of the human genome to reveal complex variations. It offers a set of efficient command line tools for various tasks, but also allows for more interactive and in-depth analysis through its integration with the Jupyter Laboratory^47^ and other data science tools. This makes it a valuable resource for researchers seeking to uncover insights from their genomic data.

The source code and library can be downloaded from https://github.com/GeneDX/pgr-tk. The documentation of the Python APIs is at https://genedx.github.io/pgr-tk/.

### SHIMMER index

SHIMMER is a data structure extending the minimizer for more efficient indexing over larger regions. Additional minimizer reduction steps to generate sparse minimizers are applied to the minimizer sequences instead of the original base-pair sequences in a hierarchical way^39^. Such sparse minimizers can serve as natural ‘anchors’ or ‘markers’ on genomics sequences without an explicit reference coordinate system. We use the SHIMMERs for quick sequence queries as initially proposed by Roberts et al.^48^. PGR-TK identifies all neighboring pairs of SHIMMERs and indexes all the sequence segments between the pairs. Figure 1b shows a cartoon of the SHIMMERs identified on each sequence. Then, the pairs of the neighboring SHIMMERs are used for indexing the corresponding sequence segments within the paired SHIMMERs. After that, we build a look-up table of all pairs of SHIMMERs to all segments with the same pair at both ends (Fig. 1c). For the query, we compute the neighboring SHIMMER pairs from a query sequence and search the database for all segments indexed by the same pairs. Finally, we can fetch all target segments stored in the database to get all related sequence information for further analysis. PGR-TK provides functions to refine the raw query results and filter out spurious alignments likely caused by repeats outside the region of interest. With the set of sequences homologous to the query sequence, we can quickly perform downstream analysis work, for example, variant discovery by aligning the sequences to each other. Furthermore, we can generate a local pangenomics (MAP-graph) for comparing the sequences in the pangenome dataset at various scales by adjusting parameters to fit different analysis tasks.

### MAP-graph

PGR-TK provides tools to generate the MAP-graph from a set of homologous sequences. The vertices in a MAP-graph are labeled with the neighboring SHIMMER pairs representing a set of sequence segments in the database (Fig. 1c,d and Methods). The edges in the MAP-graph are induced when at least one sequence connects the two fragments. Thus, each sequence naturally corresponds to a path in the graph, and the vertices in the path also contain the segments of other sequences in the database that share the same SHIMMER pair label (see the Methods for a precise mathematical definition of a MAP-graph). The successful deployment of minimizer- or minhash- based approaches in sequence comparison^39,40,49^ indicates that sequence segments with the same minimizer labels are also likely to be highly homologous. The homology between sequences can be further confirmed by explicit sequence alignment of the segment inside a MAP-graph vertex. However, the computation intensive base-to-base alignment is not required for building the MAP-graph.

The MAP-graph construction in PGR-TK is highly efficient, as it does not rely on traditional sequence-to-sequence alignment. This is demonstrated by the fast graph construction for a set of 147 MHC class II region sequences from the pangenome reference, which was completed in under 5 seconds of wall-clock time using PGR-TK, compared to the 3.5 minutes using seqwish^22^ and 13 minutes using minigraph^24^ (Supplementary Table 4).

The size of vertices in the MAP-graph, which represents the lengths of the sequence segments in the pangenome, can be adjusted by changing the parameters that determine the distance between minimizers. This allows us to study genomic features at different length scales and generate pangenome graphs with varying levels of detail. This is particularly useful when analyzing features that vary in size, such as tandem repeats in the human genome, which can range from a few hundred base pairs to 1–2 kilobases (Supplementary Table 5). By generating pangenome graphs at different levels of detail, we can gain a more comprehensive understanding of complex variation patterns within populations and focus on specific features of interest.

The analysis of pangenomic structure can be adjusted by controlling the parameters of minimizer window size (*w*), minimizer size (*k*) and hierarchical reduction factor (*r*), along with an auxiliary parameter min_span, which sets the minimum distance between minimizers in the construction of the SHIMMER index and MAP-graph (Methods). The length of each vertex in the MAP-graph, representing a sequence segment, can be modified by adjusting these parameters. This allows us to study genomics features at different length scales.

**Fig. 2:**
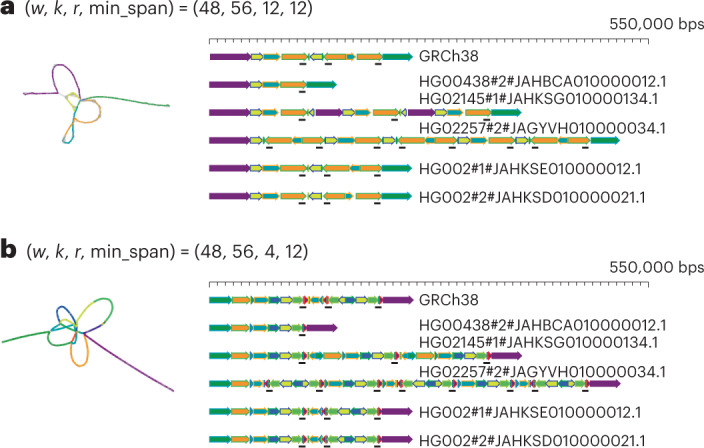
AMY1A MAP-graph in two different scales. **a**, The left panel shows a sparse MAP-graph representation of the AMY region with (*w*, *k*, *r*, min_span) = (48, 56, 12, 12). 503 vertices and 699 edges represent the 200–550 kb AMY region. The graph vertices are colored by the principal bundles that correspond to the principal bundle decomposition of selected genomes on the right panel (gray vertices are those that are not in the principal bundles). **b**, The left panel shows a denser MAP-graph with *r* = 4. The graph has 3,471 vertices and 2,684 edges, which is about 5 times as much as the MAP-graph in **a**. The principal bundle decomposition reveals a more detailed repeat structure than in **a**.

Supplementary Fig. 1 illustrates the vertex length distribution for different parameter sets using chromosome 1 of CHM13 assembly. An increase in either *w* or *r* results in longer sequences being represented by each vertex, enabling a sparser sampling of the pangenome. The choice of parameters depends on the length of the region of interest and the size of relevant biological features, such as repeat sizes and distances. For example, when studying large-scale differences, bigger *w* and *r* values are preferable to generate a sparse index that can efficiently capture large-scale differences. Conversely, to compare small-scale differences, smaller *w* or *r* values should be used. Determining the optimal parameters for the pangenome graph generation step can be challenging if the underlying interesting features are less understood. Considering this, we have found that the best initial parameter choice is determined by the length of the sequences of interest to ensure comprehensible results. Based on our observation, we provide a simple formula for selecting the parameters (Supplementary Table 6, Fig. 2 and Supplementary Fig. 2 for related examples).

### Principal bundle decomposition

A pangenome graph can serve as a cornerstone for analyzing repeat structure variation in population^17,19^. It is usually hard to compare multiple sequences with complicated repeat structure by examining pairwise sequence alignments directly. The traditional visualization technique of the dot plot^50^ allows us to perceive the complexity of the repeats but it does not provide insights into the repeat structures as linear representations across each individual sequence directly. Furthermore, only two sequences can be compared with a dot plot.

As an example, we use PGR-TK to investigate the repeat structure of the *AMY1A* gene (Alpha-amylase 1, an enzyme for the first step of catalyzing starch and glycogen in saliva) locus. We pick *AMY1A* as it has various numbers of copies caused by larger-scale structure variation related to the repeat surrounding the gene. The dot plots from randomly picking 36 sequences of a 400 kb region around *AMY1A* in the first year HPRC assemblies to the GRCh38 *AMY1A* reference sequence are shown in Supplementary Fig. 3a. Visual inspections of the dot plots show there are various numbers of copies of forward and inverted repeats forming palindromic sequences at the scales of 100 kb, and from zero up to five palindrome units. Still, only pairwise comparisons are enabled with dot plots and, thus, we lack a comprehensive assessment.

For comparison, we generate the *AMY1A* MAP-graphs at two different scales (Fig. 2) from the HPRC year one assemblies (47 samples). These can be generated with PRG-TK in less than 3 minutes from indexed sequence data. In additional to the MAP-graph, we provide tools analyzing a MAP-graph to ‘relinearize’ the graph into a set of ‘principal bundles’. We design the algorithm to generate the principal bundles representing those consensus paths that are most likely corresponding to repeat units in the pangenomes. The algorithm searches the paths that most of the pangenome sequences go through without branching as the principal bundles. This is analogous to identifying the contigs^51,52^ in genome assembly algorithms.

Figure 2 shows the principal bundle decomposition of the *AMY1A* region MAP-graph in two different scales for comparison. The smaller choice of ‘*r*’ generates a MAP-graph with more vertices in the graph and each vertex only represents a smaller portion of the pangenome. This leads to a finer scale of principal bundle decompositions.

The linear representation derived from the MAP-graph allows for efficient identification and classification of repeat structures, which are otherwise challenging to characterize. Seven genomes were selected for analysis in Fig. 2, each demonstrating distinct repeat structures. For example, HG00438 no. 2 mostly lacks repetitive sequences. The GRCh38 one has one relatively simple invert repeat (forming a palindrome region). HG02145 no. 1 has three copies of non-inverted repeats (labeled as repeat 1, 2 and 3). HG02257 no. 2 has three palindromic repeats (labeled as P1, P2 and P3). The two haplotypes from HG002 have similar structures to the GRCh38, except there is an inversion in the middle of the palindrome repeats in one of the two haplotypes. A full plot with all 96 repeat structures, including a hierarchical clustering tree identifying the similarities, is shown in the Supplementary Fig. 3b. This decomposition approach can be used by researchers to effectively classify the repeat structures of regions of interest.

### Pangenome analysis of the MHC class II locus

The MHC region in human genomes is highly polymorphic. The genomic sequences of the MHC are fundamental for understanding a human’s adaptive immune system and autoimmune diseases^53,54^. Owing to its complexity and polymorphic nature, it has been challenging to get a complete picture of the MHC genomics in the human population and to benchmark variant calling in the most variable regions^34,55^. The HPRC assemblies provide a new opportunity to analyze the MHC sequences with nearly fully assembled sequences of the region.

**Fig. 3:**
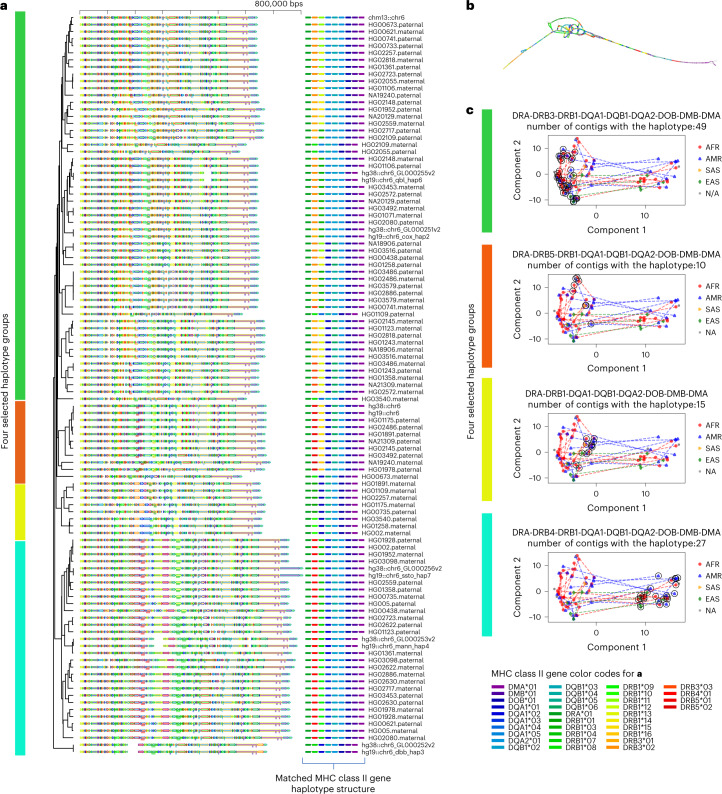
Principal bundle decomposition reveals distinct haplotype groups. **a**, The principal bundle decomposition and annotated HLA class II genes in each of the haplotype sequences. The auxiliary tracks below each sequence on the left panel show the locations of the genes. The colors of the auxiliary tracks match the gene list of genes identified for each haplotype on the right. **b**, The MAP-graph generated by PGR-TK. **c**, PCA plots of the MHC class II sequences. Each panel highlights the different gene haplotype combinations. The vertical color bars indicate the matched haplotype groups in **b** and **c**. The circled symbols indicate the haplotypes belong to the corresponding group. The dotted lines represent the connection between the two haplotypes of individuals included in the analysis set who possess both haplotypes. The population groups, African Ancestry(AFR), American Ancestry (AMR), South Asia Ancestry(SAS), East Asia Ancestry(EAS) and not applicable (NA), are indicated with different markers of different colors.

To showcase the effectiveness of PGR-TK in analyzing complicated human haplotype structures and sequences, we applied it to the HLA class II locus (GRCh38, chr6:32,313,513-32,992,088). We fetched the HPRC HLA class II haplotype sequences by anchoring them with more conserved flanking regions. Our dataset consists of a total of 105 full-length sequences, ranging from 650 to 800 kbp. Figure 3a shows the principal bundle decomposition of the collection of the sequence and Fig. 3b illustrates the MAP-graph of the 105 sequences. The tangled region in the MAP-graph represents highly polymorphic haplotypes in the human population. We can uncover the combinatorial nature of haplotype variation, such as the appearance of different combinations of HLA class II gene types in different haplotypes, in relation to the newly released human pangenome references (Fig. 3a) by generating the MAP-graph and the principal bundle decomposition of the MHC class II.

We constructed a hierarchical dendrogram on top of the principal bundle decomposition to study the relationships of highly polymorphic haplotypes in the MHC region of the human population. The PGR-TK provides a command line tool to compute a distance metric derived from pairwise sparse alignment of the bundles between two sequences and generate a dendrogram from all pairwise distances. Known HLA class II gene sequences were also mapped to 105 pangenome sequences. The full annotated principal bundle decomposition, which was annotated with HLA class II genes and the hierarchical clustering dendrogram, is shown in Fig. 3a. The highly polymorphic region in the MAP-graph was found to correspond to the *DRB-1*/*3*/*4*/*5*, *DOB* and *DOA1*/*2* region. By clustering the full set of haplotype sequences, we can identify the combination of bundles in the entire region that correspond to the gene combinations of each cluster. Our approach uses the principal bundles to classify the complete sequence, rather than relying solely on gene fragments. This classification has the potential to facilitate new applications for improved genotyping or haplotyping of larger population data in this complex region. The results could offer valuable insights into the relationship between haplotype sequences and gene combinations in the context of genetic variation and disease susceptibility.

We can also use the vertices in the MAP-graph to conduct a principal component analysis (PCA) of the MHC class II regions. We collected all vertices in the MAP-graph to form the basis of vectors. Then, we constructed a binary vector for each haplotype path by indicating whether the path of the haplotype passes through the vertex. Figure 3c displays the principal component plot of the haplotype path vectors, along with the ethnic groups. The dotted line connects the two haplotypes of an individual in the sample. We have highlighted four different groups based on the HLA class II gene combinations (excluding the subtypes). Each group contains 10–49 haplotypes. With the current dataset of a limited number of haplotypes, we have not found any statistically significant patterns yet. However, as additional data will be released from HPRC in the coming years, we anticipate that the MAP-graph can be used to systematically analyze this region and better understand its impact on human disease within ethnic group structure.

### Analyzing medically relevant amplicon genes

GIAB is using the fully assembled HG002 chrX and chrY from T2T to form new small variant and structural variant benchmarks. The assembly fully resolves the medically relevant ampliconic genes^36,56,57^ *OPN1LW*/*OPN1MW*/*OPN1MW2*/*OPN1MW3* and *DAZ1*/*DAZ2*/*DAZ3*/*DAZ4*, but the variation in these genes is too complex for current approaches to make reliable variant calls compatible with current benchmarking tools.

**Fig. 4:**
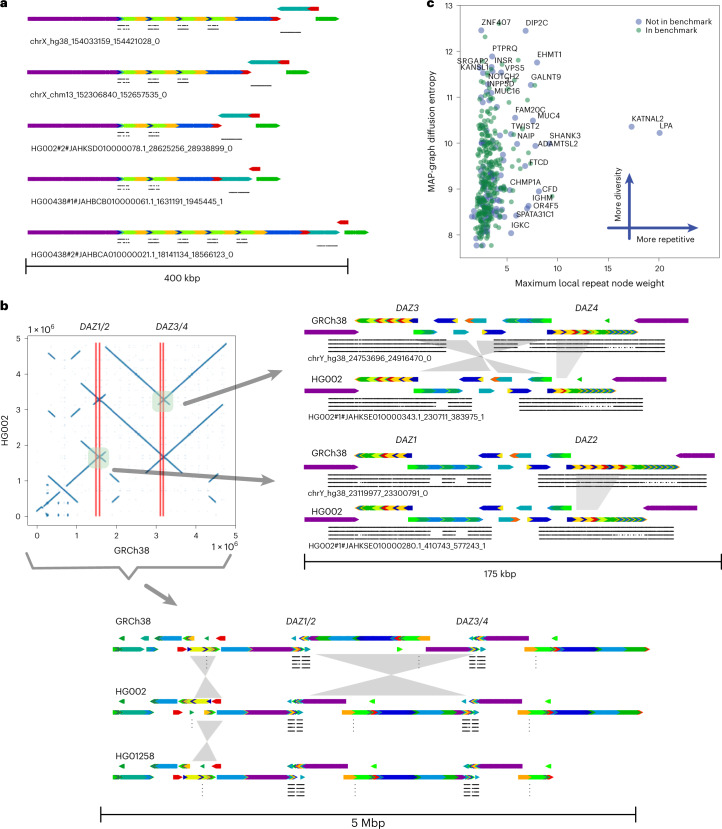
Principal bundle decomposition for genes in the repetitive regions of chromosome X and Y. **a**, MAP-graph principal bundle decomposition shows the repeat number changes of the *OPN1LW*, *OPN1MW1*/*2*/*3* to *FLNA* loci. Auxiliary tracks are as follows: top *OPN1LW*, middle *OPN1MW1*/*2*/*3* and bottom *FLNA*. **b**, The upper left image displays a dot plot comparing the HG002 assembly to GRCh38 over a 5 Mb region containing the *DAZ1*/*2*/*3*/*4* loci, highlighting an inversion between *DAZ1*/*2* and *DAZ3*/*4*. The image on the right provides a detailed view of the rearrangements at the gene scale level, with four tracks indicating the local matches to *DAZ1*/*2*/*3*/*4* from top to bottom. Comparison to GRCh38’s *DAZ2* reveals that the HG002 assembly is missing a segment (roughly 10 kb) of the darker green. The intergenic region between *DAZ3* and *DAZ4* also displays a rearrangement that can be described as an incomplete inversion or separate insertions and deletions. The bottom image shows a rearrangement at the whole locus, including all *DAZ1*/*2*/*3*/*4* over a 5 Mb region. The principal bundle decomposition reveals the different inverted structure of the HG002 T2T assembly and the HG1258 assembly compared to GRCh38. **c**, MAP-graph diffusion entropy versus repetitiveness survey for the 385 GIAB challenge CMRGs.

For example, the genes *OPN1MW* and *OPN1MW2* are inside a 74 kb deletion in HG002 relative to GRCh38, so HG002 contains only two of the four copies of the array in GRCh38: *OPN1LW* and one copy of *OPN1MW*/*OPN1MW2*/*OPN1MW3*. Dipcall^58^ can call the 74 kb deletion and variants in the other gene copies, but it may be possible to align the assembly to GRCh38 in alternative ways. The visualization from PGR-TK makes clear the varying number of genes in this array in each haplotype in Fig. 4a, which is important for some phenotypes such as color blindness, since seeing full color requires *OPN1LW* and at least one copy of *OPN1MW*/*OPN1MW2*/*OPN1MW3* (refs. ^56,59^).

Another important gene family *DAZ1*/*DAZ2*/*DAZ3*/*DAZ4* are in a set of nested palindromic repeats. It has been reported that partial deletions in this region may cause male infertility^57^. It would be useful to understand the natural distribution of non-pathogenic structural variants across this ampliconic gene cluster. *DAZ1* and *DAZ2* are roughly 1.5 Mbp (megabasepairs) from *DAZ3* and *DAZ4*, and HG002 has a 1 to 2 Mbp inversion relative to GRCh38 with breakpoints in the segmental duplications that contain the *DAZ* genes (Fig. 4b). In addition to the large inversion, the *DAZ* genes contain structural variants, including a roughly 10 kb deletion in *DAZ2*, two deletions in *DAZ4* and two insertions in *DAZ3* of sequences that are only in *DAZ1* and *DAZ4* in GRCh38. PGR-TK’s ability to color and visualize variation with the principal bundle decomposition algorithm at multiple scales enables intuitive understanding of this type of very complex variation, which would be very difficult to represent and understand as simple structural variant calls in VCF format.

The efficiency of PGR-TK makes it suitable for analyzing complex variants at isolated loci, as well as a set of region of interest. For example, GIAB has identified a set of 395 challenging but medically relevant genes. Analyzing this set in the pangenome references will provide some insight into the related complicated variation at the population level.

Using PGR-TK, we extracted all sequences of the 395 genes from the HPRC year one release (94 haplotype assemblies), CHM13 v.1.1, GRCh38 and hg19 of all 385 CMRG. We generated a MAP-graph for each gene and output it in GFA format (as seen in the Supplementary Fig. 4). For each graph, we derived two metrics to estimate (1) the degree of polymorphism among the pangenomes, and (2) the repeat content considering the variations of the pangenomes (as shown in Fig. 4c). These two measurements provide independent assessments of the MAP-graph structures of these genes. We found, as expected, that highly repetitive genes (such as *LPA* and *KATNAL2*) are more difficult to create a reliable variant benchmark call set. Many highly repetitive genes are excluded from the current CMRG benchmark set. We did not observe a correlation between higher entropy and the reduction of the gene in the benchmark set. We found that the high entropy genes also span larger regions in the genome. While entropy can indicate the complexity of variations in the population, we observed different clustering structures of the top entropy genes (see the comparison of the MAP-graph PCA plots of *SNTG2* and *KMT2C* in Supplementary Fig. 5).

## Discussion

With the advance in DNA sequencing technologies, more comprehensive human genomes at, or close to, telomere to telomere will be collected and made available in the coming years. It will enable researchers to study and characterize those previously inaccessible complex, but likely relevant, regions. The current HPRC assembly release has significantly affected our understanding of the human genome architecture. It will also be essential for building applications for clinical and medical tests and diagnostics soon. Flexible and scalable computational tools for analyzing pangenome-level genome assemblies will be part of the vital task of improving the practice of precision medicine with rich genomic data such as those from HPRC.

Many of the recently developed pangenome analysis tools allow graph analysis at the whole-genome level^22–24^. Meanwhile, the richness of diversity of human genomes over the repetitive regions poses unique challenges for analysis. In our work developing PGR-TK, we focus on providing a flexible library of useful algorithms. Furthermore, it enables analyzing the genome assemblies such that a developer or a researcher can rapidly access certain complex regions by adjusting parameters for visualization and integrating with subsequent analysis. PGR-TK may not be suitable for analysis at the whole-genome scale yet, and obtaining optimized results with the tool might not be straightforward. The challenges associated with the diversity of human genomes over repetitive regions can complicate the analysis process. It is crucial to ensure that the parameters chosen for visualization and subsequent analysis are appropriate for the specific genomic characteristics being examined. It may require careful consideration and expertise of a user to fine-tune the parameters and algorithms for optimization. Each main building unit (the vertex) of the MAP-graph represents a set of closely related sequence fragments. This is more analogous to the stringomics method proposed by Ferragina^21^ than other methods building graphs on top of MSA or variant calls. Such approaches combined with the sparse minimizers are efficient to reduce the computational complexity (fewer vertices) to represent larger-scale scale structures. Complementary to that, PGR-TK provides an interface to fetch the sequences within each vertex such that it is possible to combine a MAP-graph with other graph analysis approaches. For example, it can be integrated with Cactus graph^25^ and A-de Bruijn Graph^42^ for base-level analysis, such as variant calling, genotyping and point mutation analysis, with recursive hybrid graph data structures.

We demonstrate how to use the PGR-TK for studying and characterizing the repetitive region *AMY1A* and the highly polymorphic HLA class Il region. We present a tool in PGR-TK backed by a new pangenome graph traversal algorithm, relinearizing tangled graphs caused by repetitive sequences to principal bundles for visualization. With the principal bundle decomposition, we can automatically visualize the repetitive and non-repetitive components of haplotype assembly contig. The PGR-TK can provide intuitive qualitative information about different genome arrangement architectures with decomposition and associated visualizations. For example, it enables visualization of both the very large inversion in the *DAZ* locus and much smaller complex structural variation within the genes. The visualization of the *OPN1LW*/*OPN1MW* gene array shows subtle copy number variations, which can affect vision, as well as nearby structural variants. We also use PGR-TK to survey a set of regions of interest across the whole genome. We derive two metrics for measuring the polymorphism and repetitiveness in human pangenome to more systematically survey complexity of a large set of medically and clinically relevant genes. In the future, we aim to extend the PGR-TK library to provide more quantitative and base-level analysis for both fundamental and translational research using pangenome resources.

## Methods

### Sequence database and SHIMMER index construction

To generate the SHIMMER index, each sequence was scanned and the symmetrical minimizers were generated with the specific minimizer window size *w* and kmer size *k*. We called this the first level of minimizer. Given a reduction factor *r* > 1, additional levels of minimizer sets were generated to increase the span between the minimizers by a reduction step to facilitate pangenomics analysis. Even with the reduction step, in some simple sequence context, for example, long or short tandem repeats, two minimizers can remain too close to each other. A parameter ‘min_span’ can be applied to eliminate a pair of minimizers that are too close. We used a heuristic algorithm to eliminate those minimizers that were within the distance of min_span to each other. This helped to reduce the minimizer density when detailed analysis for those simple context regions was not desired. Setting min_span to zero and *r* to one generated the standard minimizers for each sequence.

Each pair of the reduced minimizers (SHIMMERs) was used as the key to build a hashmap to the sequence ID, and coordinates and matching orientation of the minimizer pairs on the sequences.

We also present examples of API calling and command line usage below.

### Generate MAP-graph

The MAP-graph is constructed by scanning through each sequence in the database. The vertices are simply the set of the tuples of neighboring minimizers (minimizer anchored segments). The edges are constructed by connecting minimizer anchored segments as a bidirected graph. One can consider this to be an extension of the string graph^52^ where the overlaps are the minimizers at both ends. However, in the pangenome graph, each vertex includes a set of sequence segments from multiple genomes rather than one sequence.

As the MAP-graph can be constructed by scanning the SHIMMER pairs through the sequences. For a given set of *n* sequences, *S* = {*s*_*i*_|*i* = 0…*n* − 1}, the vertices of the MAP-graph are

$$\begin{array}{l}V = \left\{\right.({m^{(i)}}_{p},{m^{(i)}}_{p+1})|{m^{(i)}}_{p}, {\mathrm{and}}\, {m^{(i)}}_{p+1}\, {\mathrm{are}}\, {\mathrm{the}}\, p{\mathrm{th}}\, {\mathrm{and}} (p + 1){\mathrm{th}} \\\qquad{\mathrm{minimizers}}\, {\mathrm{of}}\, {\mathrm{a}}\, {\mathrm{sequence}}\, s_{i}, {\mathrm{in}}\, {\mathrm{all}}\, s_{i}\, {\mathrm{in}}\, S\left.\right\}.\end{array}$$

We can assign a weight *w*_*i*_ of a vertex *v*_*i*_ = (*m*_a_, *m*_b_) as the total number of observed (*m*_a_, *m*_b_) pairs in *S*.

The edges of the MAP-graph are

$$\begin{array}{l}E = \left\{\right.(v_{i}, v_{j})|v_{i} = ({m^{(i)}}_p,{m^{(i)}}_{p+1})\, {\mathrm{and}}\, v_{i} = ({m^{(i)}}_{p+1},{m^{(i)}}_{p+2})\, {\mathrm{for}}\, {\mathrm{all}}\\\qquad ({m^{(i)}}_p, {m^{(i)}}_{p+1},{m^{(i)}}_{p+2})\, {\mathrm{in}}\, {\mathrm{all}}\, s_{i}\, {\mathrm{in}}\, S\left.\right\}\end{array}$$

### Identify the principal bundles in a MAP-graph

To decompose a MAP-graph into principal bundles for downstream analysis, we apply a variation of depth first search^60^ (DFS) to build the traversal trees from the graph. Our DFS prioritized vertices with high ‘weight’ (defined as the number of sequence segments contained in a vertex) and accounting for the bidirected nature of the MAP-graph.

The DFS traversal through the graph is then converted to a tree structure internally. The leaf nodes in the tree are typically when the DFSs are terminated by no out edge from a node or a bubble or a loop is found. As we prioritize the weights of the vertices during DFS, long paths usually correspond to the ‘common’ paths that most sequences in the data would go through. Rarer haplotypes typically correspond to short bubble paths in the MAP-graph. Thus, they can be identified as short branches in the DFS traversal tree. We use the tree to remove those vertices in the MAP-graph if those are shorter than prespecified length in the DFS tree. In general, the vertices in the principal bundle represent more sequences in the set of input sequences (Supplementary Fig. 6) and are likely more conservative in the pangenome.

After removing the vertices corresponding to the short branches, we further remove vertices in the MAP-graph that have more than three out edges after converting the MAP-graph as an undirected graph. After such removal, the graph will only consist of simple paths, and we output those paths as the principal bundles.

In summary, here is the sketch of the algorithm:

1. Build a DFS traversal tree with a deep first search for a given MAP-graph. To capture paths that are more conserved among the pangenome sequences of interest as the principal bundles, our DFS search prioritizes high-weight vertices when constructing the DFS traversal tree
2. In MAP-graph, remove vertices that correspond to nodes in short branches of the DFS traversal tree
3. Remove branching vertices in MAP-graph (by considering it as an undirected graph)
4. Output the simple paths from the resulting graph as the principal bundles.

### Principal component plot for the HLA class II locus

To generate the principal component of the pangenome HLA class II sequences, we convert each of the haplotype sequences to a binary vector. The binary vector has the same length of the total number of vertices of all principal bundles. Let us call these vertices *V* = {*v*_*i*_|*v*_*i*_ in principle bundles, *i* = 0_*n*−1_}, where *n* is the total number of vertices in the principal bundles. For each sequence *s*, we construct a binary vector *w*_*s*_ = {*b*_0_, *b*_1_,…, *b*_*n*−1_} where *b*_*i*_ = 1 if the sequence *s* contains the vertex *v*_*i*_, and *b*_*i*_ = 0 if not. Then, we perform the standard principal component transformation with the binary vectors of all sequences from the HLA class II region.

### General workflow for analyzing a region of interest

Here we outline the general workflow on how to use PGR-TK to generate MAP-graph and the principal bundle decomposition.

1. For a region or sequences of interest, put the sequences as a fasta file for querying the PGR-TK pangenome sequence database. (PGR-TK provides a command line tool ‘pgr-fetch’ and python APIs to fetch such sequence from the PGR-TK sequence database.)
2. Query the whole pangenome database to get initial hits that match the query sequence with the command line tool ‘pgr-query’ or using the Python APIs.
3. Filter the hits to remove unwanted matches that do match a user’s analysis objectives. With the command line tool ‘pgr-query’, it generates a summary table of the hits for filtering.
4. With the filtered sequences, using ‘pgr-pbundle-decomp’ command line tool to generate the MAP-graph in GFA format and principal bundle decomposition in the BED format. Beside analyzing the generated data, the generated bed file of the decomposition can be rendered by the pgr-pbundle-bed2svg to generate visualization. Python APIs are also provided for more scripting to resolve complicated analysis cases.
5. Optionally, we can take the fetched sequences from ‘pgr-query’ to use with other third party tools, for example, calling variant with dipcall, create an MSA, or building other local pangenome graphs with minigraph or pggb.
6. Re-adjust the parameters (*w*, *k*, *r*, min_span) and repeat (3) and (4) with additional analysis of the results if necessary.

### Building index

For a large sequence set, for example 47 whole-genome HPRC assemblies, PGR-TK uses the AGC format^46^ to store the sequence efficiently. A command line tool ‘pgr-mdb’ is developed with the PGR-TK package to create the index file on top of the AGC file. For example, for a prebuild HPRC year one assembly AGC file (1.33 Gb), we create a file (/data/pgr-tk-HGRP-y1-evaluation-set-v0_input) include a file system path to the AGC file, /data/gr-tk-HGRP-y1-evaluation-set-v0.agc and call ‘pgr-mdb‘ to create the index files with a prespecified prefix (/data/pgr-tk-HGRP-y1-evaluation-set-v0):


echo /data/pgr-tk-HGRP-y1-evaluation-set-v0.agc \> /data/pgr-tk-HGRP-y1-evaluation-set-v0_input/code/pgr-mdb /data/pgr-tk-HGRP-y1-evaluation-set-v0_input \/data/pgr-tk-HGRP-y1-evaluation-set-v0


Two files will be generated in this example


/data/pgr-tk-HGRP-y1-evaluation-set-v0.mdb # 15 Gb for (w, k, r, min_span) = (80,56,4,64)/data/pgr-tk-HGRP-y1-evaluation-set-v0.midx # 3.1 Mb


The index and sequence data can be loaded into a python workspace by


import pgrtksdb = pgrtk.SeqIndexDB()sdb.load_from_agc_index(‘pgr-tk-HGRP-y1-evaluation-set-v0’)


As the indexes are loaded into memory, we suggest using a computing instance that has a random access memory larger than about four times that of the index file to avoid swapping thrashing.

For smaller sequence files, the sequence database object (for example, the ‘sdb’ in the example above) created by pgrtk.SeqIndexDB()can create and load sequences using load_from_fastx() method. See the library documentation at https://genedx.github.io/pgr-tk/ for more detailed descriptions of all python objects, methods and functions in the PGR-TK package.

### Query sequence in the PGR-TK sequence database

#### Command line example

A command line tool named pgr-query is provided to query a PGR-TK database with a set of sequences, each of which represents a region of interest. It is recommended to select regions larger than 20 kb, as they contain enough SHIMMER anchors. If smaller regions are of interest, padding with flanking sequences can improve the results.

The follow command shows an example querying the database: pgr-query /data/pgr-tk-HGRP-y1-evaluation-set-v0 ROI_seq.fa pg_seqs --merge-range-tol 100,000.

In this example, the ROI_seq.fa file contains sequences from the regions of interest. The pgr-query tool generates a set of fasta files with the prefix ‘pg_seqs’ for each query sequence in the ‘ROI_seq.fa’ file. Additionally, a ‘pg_seqs.hit’ file is produced, which contains information about the alignment range between the query and the results, as well as the number of anchors identified between each pair of query results. This information can be used to filter out unwanted alignments.

#### Python API example

For finding homologous sequences in a PGR-TK database, we need to start with a query sequence. We can fetch a sequence in the database giving a known ‘data source’, ‘contig name’ tuple and the beginning and ending coordinates. As the SHIMMERs are sparsely distributed in a sequence, the query sequence should be long enough to cover enough minimizer anchors. The python statement shows a typical code fragment to generate query results of a region of interest:


ref_file_name, roi_chr, roi_b, roi_e = ‘hg19_tagged.fa’, ‘chr6_hg19’, 32130918, 32959917padding = 10000#get a segment of a referenceroi_seq = ref_db.get_sub_seq(ref_file_name, roi_chr, roi_b-padding, roi_e+padding)# using the roi_seq to find hits in ‘sdb’aln_range = pgrtk.query_sdb(sdb, roi_seq, merge_range_tol=200000)


The output aln_range from the query_sdb() call contains data of the hits in the PGR-TK database. Internally, the query_sdb() method performs:

1. create SHIMMER pairs of the query sequence (2) use the SHIMMER pairs and the hashmap index to find all hits in the database
2. perform sparse dynamic programming to find sparse alignments between the query sequence and all hits in the database
3. merge the alignment segments if any of them are within the merge_range_tol parameter.

The parameter merge_range_tol is introduced to avoid alignment fragmentation when the query sequence contains a region of high polymorphism but we still want to fetch those diverse sequences for constructing the pangenomics graph.

Typically, a user needs to process the data in aln_range for different analysis. Our example Jupyter Notebooks provides various examples for processing the output to generate dot plot or MAP-graphs and so on.

### Build minimizer anchor pangenome graph and principal bundle decomposition

#### Command line example

Given a set of sequence in a fasta file, for example, the query results from pgr-query command, we can build the pangenome graph and the principal bundle decomposition (outputted as a bed file) by


pgr-pbundle-decomp -w 48 -k 56 -r 8 \--min-span 12 --bundle-length-cutoff 100 \--bundle-merge-distance 1000 --min-branch-size 8 \--min-cov 0 --include file_contain_contig_names \pgr-query pgr_out


In the pangenome graph construction process, the following options can be used to control the graph construction: -w, -k, -r and --min-span. The --min-cov option sets the minimum coverage requirement for a vertex to be included in the principal bundle graph. The --min-branch-size option allows the user to filter out short branches that contain less than a specified number of vertices in the MAP-graph. The --bundle-length-cutoff option allows the user to exclude bundles shorter than the specified length. When two bundles have the same identifier and are within the distance specified by --bundle-merge-distance, they will be merged. If the --include option is specified, only those contigs specified in the file_contain_contig_names file will be analyzed.

The command generates a set of files with the prefix pgr_out:


pgr_out.bed # the bed file contains the principal bundle decompositionpgr_out.ctg.summary.tsv # summary for bundle statistics for each contigpgr_out.mapg.gfa # MAP-graph in GFA formatpgr_out.mapg.idx # index for the sequence in the GFA filepgr_out.pmapg.gfa #principal bundle graph in the GFA format


The pgr_out.bed has the following format. Each line is a bundle contain in a contig: contig_name begin_coordinate end_coordinate bundle_specification where the bundle_specification is six fields delimited by ‘:’. The fields are bundle identifier, bundle vertex count, orientation, begin vertex number, end vertex number, ‘R’ or ‘U’ for repetitive or unique in the contig. Note such a region in a contig may not only project to the full bundle. In such cases, end_vertex-bgn_vertex is less than bundle_vertex_count.

The *.mapg.idx file contains the information about each vertex in the .gfa files. It contains three kinds of tagged line starting with ‘K’, ‘C’ and ‘F’. The K line specifies the parameters used to generate the graph. The C lines specify the contigs contained in the graph. The F lines specify the fragment of sequences contained in each vertex. Here are the fields for echo of them: K line: *w*, *k*, *r*, min_span

S line: contig_identifer, contg_name, contig_source, contig_length

F line: frag_unique_identifier, frag_numeric_identifer, contig_identifer, start_coordinate, end_coordinate, orientation

Python API:

To build the MAP-graph and principal bundle decomposition within a python program, one can use the instance methods generate_mapg_gfa() and get_principal_bundle_decomposition() of an pgrtk.SeqIndexDB() object. Please read the documentation at https://genedx.github.io/pgr-tk/ for the API details.

### Clustering principal bundle decomposition structure

The follow command performs clustering of the principal bundles stored in a bed file: pgr-pbundle-bed2dist pgr_out.bed pgr_out.

It will generate these output files: pgr_out.dist # all pairwise distance


pgr_out.nwk # a hierarchical tree in Newick formatpgr_out.ddg # a file contain the dendrogram for the pgr-pbundle-bed2svg to draw the dendrogram panel


### Generate principal bundle decomposition plot in scalable vector graph format

The following is an example to generate a SVG file pgr_out.svg from pgr_out.bed that layout 500,000 bp with annotation specified in a file called pgr_annotation with a dendrogram panel on the left from the pgr_out.ddg dendrogram data


/code/pgr-pbundle-bed2svg pgr_out.bed pgr_out \--track-range 500000 --track-tick-interval 100000 \--track-panel-width 1200 --stroke-width 0.5 \--annotations pgr_annotation \--ddg-file pgr_out.ddg \--highlight-repeats 3


### Survey on the genome in bottle challenging clinical and medically relevant genes with the MAP-graphs

The GIAB Consortium provides variant call benchmarks on seven benchmark genomes^35,61,62^. These benchmarks were initially formed by integrating multiple short-read technologies, but the latest version integrated linked-read and long-read technologies to form benchmarks in regions difficult to map with short reads. However, a set of 395 challenging but medically relevant genes were identified as substantially (more than 10%) excluded from the mapping-based benchmark due to long repeats, large structural variations, segmental duplications and/or high polymorphism between the benchmark genome HG002 and the reference genome hg19 or GRCh38. A long-read genome assembly approach provided a reliable benchmark call set of 273 out of the 395 genes^35^. The remaining 122 were still excluded mainly because they were not accurately assembled, or no benchmarking tools exist to compare different representations of complex variants in the genes. Overall, 395 genes have recently shown to include high levels of polymorphism across different ethnicities making it highly challenging to represent their variations^63^.

The current GIAB variant benchmarks focus on a small set of seven well-characterized genomes with extensive short-, linked- and long-read data to ensure robust benchmarks and more tractable method development and benchmark evaluations. Meanwhile, a limited representation of genomes in a population may miss significant structural variants, additional copies of genes and context for important variants for diseases not observed in a smaller dataset. Given that increasingly accurate long-read and assembly level data are being produced at pangenome scales now, we are surveying how we can use such resources to benchmark variant accuracy at a broader population level for the challenging CMRG. Such pangenome analysis will help to generate guidelines for future practice.

With PGR-TK, we extract all sequences from the HPRC year one release (94 haplotype assemblies), and CHM13 v.1.1, GRCh38 and hg19 of all 385 CMRG. We generate a MAP-graph of each gene and output this in GFA format. For each graph, we derived two metrics to estimate (1) the degree of polymorphism among the pangenomes, and (2) the repeat content taking account of the variations of the pangenomes.

To estimate the degree of polymorphism, we consider a diffusion process in the graph (below) and derive an entropy-like quantity from a normalized equilibrium distribution. The higher entropy values indicate more complicated graphs likely from the polymorphism from different genomes. The diffusion weight on each vertex is also associated with multiplicity and repetitiveness of the corresponding segments in the pangenomes. We pick the average of the top 32 diffusion weights from the MAP-graph of each gene as a simple metric to measure the most challenging repeat content within a gene.

To gain insight about the challenge for calling variants of the CMRG set at a pangenome scale, we plot the diffusion entropy versus the maximum local repeat weights for each gene (Fig. 4c). As there are no obvious correlations, these two quantities provide independent measurements of two aspects of the MAP-graph structures of these genes. We find high repetitive genes are harder to create a reliable variant benchmark call set for. Many highly repetitive genes are excluded from the current CMRG benchmark set. We do not observe that higher entropy is correlated with the reduction of the gene in the benchmark set.

We highlight several genes with high entropy or high repetitiveness. The MAP-graphs and the Integrative Genomics Viewer view of the pangenome assemblies of a selected set genes (*LMF1*, *ANKRD11*, *SRGAP2*, *KMT2C*, *LPA*, *MUC4*, *MUC3A*, *KATNAL2*, *FLG*) aligned to GRCh38 are shown in Supplementary Fig. 4. In the Integrative Genomics Viewer view of *LMF1* (Supplementary Fig. 4a), several variation hotspots are visible and may correspond to localized structural variants. This serves as a simple example to examine the concordance of structural variation in the population and the principal bundle decomposition. We use PAV^64^ to call structural variants for comparison. In Supplementary Fig. 4b, the structural variant calls resulting from PAV are provided as an auxiliary track (black) below the principal bundle decomposition tracks for a selected set of HPRC year one genomes, where both haplotypes are resolved in the region. It is evident that the structural variation calls correspond to the regions where principal bundles have complex structure and are distinct from the reference genome GRCh38 (the top track). As shown in *LPA* (associated with coronary disease^65^, Supplementary Fig. 7), *KATNAL2* (loss of function variant discovered in autistic proband^66,67^) also has long tandem repeat variations in the HPRC pangenome cohort. We find the number of the 5.8 kbp repeats inside *KATNAL2* ranges from 3 to 25 (Supplementary Fig. 8). Applying MAP-graph decomposition on genes such as *KATNAL2* with a big pangenome reference panel will provide additional insights to the natural of the variability of the repetitiveness and its effects on the underlying biology like other, more well-studied genes, for example, *LPA*, in the coming years.

### Compute graph diffusion entropy and max repetitive weight

It would be desirable to derive quantitative measurements so we can characterize a set of large numbers of MAP-graphs fast. One thing we are interested in quantifying is how complex a graph is. The intuition is that if a region of the genome is more polymorphic in the population, the graph will have more alternative paths, or bubbles. We like to generate a quantity as a proxy for that. For this, we borrow the idea from network science study and spectral graph theory to consider a diffusion/random walk process on a graph^68^. For a graph, we consider a set of random walkers starting at each vertex. The random walkers can drift on the graph through the edge-connection. We can consider the distribution of the random walkers in the final equilibrium state. If a graph is relatively simple, then the final distribution will be uniform (subject to minor boundary condition corrections). On the other hand, if the weights of the vertices or topology of the graph are more complex, we would expect the final distribution of the walkers would be less uniform and reflect the complicated nature of the graph.

The final distribution of the such diffusion process can be obtained by simple matrix multiplication iteration from the adjacent matrix of a MAP-graph. Given an adjacency matrix *A*, where the matrix element *A*_*ij*_ is the number of sequences supports edge from *v*_*i*_ to *v*_*j*_. The final distribution *P* can be written as *P* = (1/*N*) lim_*n*→∞_ *M*^*n*^ **1**, where *M* = *AD*^−1^, *D* is the degree matrix defined as *D*_*ii*_ being the degree of vertex *v*_*i*_ and *D*_*ij*_ = 0 if *i* ≠ *j*, *N* is the total number of vertices and **1** is a column vector in which every element is one. (When we compute *P*, we only repeat the number of multiplications *N* times to approximate the final distribution.)

**P** is a normalized column vector [*p*_0_, *p*_1_,…,*p*_*n*−__1_]^*T*^ such that ∑_*i*=0…*n*−1_*p*_*i*_ = 1 (see Supplementary Fig. 4a for an example). The diffusion entropy used in this work is defined as *S* = −∑_*i*=0…*n*−__1_ *p*_*i*_ log_2_(*p*_*i*_).

To find the highly repetitive elements inside a region of interest, we look into largest elements in the unnormalized vector *N***P** as a proxy of average number of repeats considering the graph structure. We pick the top 32 elements in *N***P** and use the average of those as a proxy number to estimate the repetitiveness of potential repeat units inside a region of interest.

### Reporting summary

Further information on research design is available in the Nature Portfolio Reporting Summary linked to this article.

## Online content

Any methods, additional references, Nature Portfolio reporting summaries, source data, extended data, supplementary information, acknowledgements, peer review information; details of author contributions and competing interests; and statements of data and code availability are available at 10.1038/s41592-023-01914-y.

## Supplementary information

Supplementary Information Supplementary Tables 1–6 and Figs. 1–8.

Reporting Summary

## Data Availability

The HPRC year one release sequence and prebuilt index: https://giab-data.s3.amazonaws.com/PGR-TK-Files/pgr-tk-HGRP-y1-evaluation-set-v0.tar and https://figshare.com/articles/journal_contribution/HPRC-Y1-sequence-data_zip/22584403 (10.6084/m9.figshare.22584403). Scripts and source data URLs for constructing the HPRC AGC file: https://github.com/GeneDX/pgr-tk-notebooks/tree/main/pgr-tk-sequence-source. All GFA files, fetched sequences from HPRC year one release of the 385 CMRG: https://giab-data.s3.amazonaws.com/PGR-TK-Files/CMRG_output_dir_v0.3.3.tar.

## Code availability

The code of the PGR-TK is hosted in a GitHub repository, https://github.com/GeneDX/pgr-tk, and the code can be obtained using the standard ‘git clone’ command. Follow the README.md document for building the PGR-TK. A document for Python API is provided at https://genedx.github.io/pgr-tk/. The source code for the v.0.4.1 version is also hosted at https://figshare.com/articles/journal_contribution/pgr-tk-0_4_1_zip/22584793 (10.6084/m9.figshare.22584793). Prebuilt binaries for Linux Ubuntu distribution 22.04 can be downloaded from https://github.com/GeneDX/pgr-tk/releases/tag/v0.4.1 and https://figshare.com/articles/journal_contribution/pgr-bin-pacakge-v0_4_1_tgz/22584787 (10.6084/m9.figshare.22584787). Example Notebooks using PGR-TK including code making most of (the source of) the plots in this paper can be found at https://github.com/GeneDX/pgr-tk-notebooks and https://figshare.com/articles/journal_contribution/scripts_and_artifects_zip/22584118 (10.6084/m9.figshare.22584118) Additional information about a docker image with prebuilt PGR-TK library and Jupyter Laboratory Server and usage is at https://github.com/GeneDX/pgr-tk/blob/main/pgr-tk-workstation/Readme.md.
