# Supplementary material for: Multiscale analysis of pangenomes enables improved representation of genomic diversity for repetitive and clinically relevant genes
Source: Nat Methods. 2023 Jun 26;20(8):1213–21. doi: 10.1038/s41592-023-01914-y (PMC10406601; doi:10.1038/s41592-023-01914-y)

nature methods

Article https://doi.org/10.1038/s41592-023-01914-y

Multiscale analysis of pangenomes enables improved representation of genomic diversity for repetitive and clinically relevant genes

In the format provided by the authors and unedited

Supplementary Material

Table of Contents

Performance Evaluation **2**
Sequence Query 2
Supplementary Table 1 2
Supplementary Table 2 4
Supplementary Table 3 6
Effect of the Parameter Choice to The MAP Vertex Sizes 10
Supplementary Figure 1 10
Supplementary Table 5 12
Suggested Parameter Choice for Region of Size Up to 5Mb 13
Supplementary Table 6 13

Generate MAP-Graph and Principal Bundle Decomposition for AMY and MHC regions **14**
Comparing Principal Bundle Decomposition with Different Set of Parameters 15
Supplementary Figure 2 17
AMY1A repeat dot plots and principal bundle decomposition plots 18
Supplementary Figure 3 18

GIAB CMRG cases **20**
Supplementary Figure 4 20

SNTG2 and KMT2 **30**
Supplementary Figure 5 30

The distribution of the vertex weight on the principal bundle vertices for MHC class II, *AMY1A* and *LPA* **32**
Supplementary Figure 6 32

LPA, KIV-II repeats principal bundle decomposition plot **33**
Supplementary Figure 7: LPA, KIV-II repeats principal bundle decomposition plot 33

Principal bundle plot for KATNAL2 **34**
Supplementary Figure 8 34

Performance Evaluation

Sequence Query

While PGR-TK is not designed for creating sequence alignments, the query sequence to database query provided function to identify homologuos sequences in the database to the query sequences. We compare the computing resource for such utility in PGR-TK to minimap2, currently the state of are for fast sequence alignment. For a set of ten selected regions for querying 11 haplotype pangenome references, PGR-TK can index all genome in parallel and provide comparable query time.

Supplementary Table 1

	Tool	
**Computation Resources**	**pgr-tk**	**minimap2**
index time (elapsed time)	6 min 43 sec (agc database building = 2 min 59 sec and indexing = 2 min 44 sec)	13 min 07 sec
query time	8.45 sec (including fetching the sequences)	17.93 sec
sequnece storage	807 Mb	9.0 Gb
index storage	2.0 Gb	75.9 Gb

Our testing pangenome dataset contain the assemblies from the HGRP samples:

```
HG00438.maternal, HG00438.paternal, HG00621.maternal, HG00621.paternal,
HG00673.maternal, HG00673.paternal, HG00735.maternal, HG00735.paternal,
HG00741.maternal, HG00741.paternal
```

minimap2:
source: https://github.com/lh3/minimap2
revision: `01b98e8e52a8acfed5a9d57853f028267eaf045f`

commands:

Minimap2 index:

```
\time -v ./minimap2/minimap2 HG00438.maternal.f1_assembly_v2_genbank.fa.gz -t 16 -d
HG00438.maternal.f1_assembly_v2_genbank.fa.gz.idx &>> minimap_timing1.log
\time -v ./minimap2/minimap2 HG00438.paternal.f1_assembly_v2_genbank.fa.gz -t 16 -d
HG00438.paternal.f1_assembly_v2_genbank.fa.gz.idx &>> minimap_timing1.log
```

```
\time -v ./minimap2/minimap2 HG00621.maternal.f1_assembly_v2_genbank.fa.gz -t 16 -d
HG00621.maternal.f1_assembly_v2_genbank.fa.gz.idx &>> minimap_timing1.log
\time -v ./minimap2/minimap2 HG00621.paternal.f1_assembly_v2_genbank.fa.gz -t 16 -d
HG00621.paternal.f1_assembly_v2_genbank.fa.gz.idx &>> minimap_timing1.log
\time -v ./minimap2/minimap2 HG00673.maternal.f1_assembly_v2_genbank.fa.gz -t 16 -d
HG00673.maternal.f1_assembly_v2_genbank.fa.gz.idx &>> minimap_timing1.log
\time -v ./minimap2/minimap2 HG00673.paternal.f1_assembly_v2_genbank.fa.gz -t 16 -d
HG00673.paternal.f1_assembly_v2_genbank.fa.gz.idx &>> minimap_timing1.log
\time -v ./minimap2/minimap2 HG00735.maternal.f1_assembly_v2_genbank.fa.gz -t 16 -d
HG00735.maternal.f1_assembly_v2_genbank.fa.gz.idx &>> minimap_timing1.log
\time -v ./minimap2/minimap2 HG00735.paternal.f1_assembly_v2_genbank.fa.gz -t 16 -d
HG00735.paternal.f1_assembly_v2_genbank.fa.gz.idx &>> minimap_timing1.log
\time -v ./minimap2/minimap2 HG00741.maternal.f1_assembly_v2_genbank.fa.gz -t 16 -d
HG00741.maternal.f1_assembly_v2_genbank.fa.gz.idx &>> minimap_timing1.log
\time -v ./minimap2/minimap2 HG00741.paternal.f1_assembly_v2_genbank.fa.gz -t 16 -d
HG00741.paternal.f1_assembly_v2_genbank.fa.gz.idx &>> minimap_timing1.log
\time -v ./minimap2/minimap2 chm13.draft_v1.1.fasta.gz -t 16 -d chm13.draft_v1.1.fasta.gz.idx
&>> minimap_timing1.log
```

Minimap2 query:

```
cat << EOF | \time -v parallel -j 16 2> minimap_timing.log 1> minimap.hits
./minimap2/minimap2 -x asm5 HG00438.maternal.f1_assembly_v2_genbank.fa.gz.idx ROI_seq.fa
./minimap2/minimap2 -x asm5 HG00438.paternal.f1_assembly_v2_genbank.fa.gz.idx ROI_seq.fa
./minimap2/minimap2 -x asm5 HG00621.maternal.f1_assembly_v2_genbank.fa.gz.idx ROI_seq.fa
./minimap2/minimap2 -x asm5 HG00621.paternal.f1_assembly_v2_genbank.fa.gz.idx ROI_seq.fa
./minimap2/minimap2 -x asm5 HG00673.maternal.f1_assembly_v2_genbank.fa.gz.idx ROI_seq.fa
./minimap2/minimap2 -x asm5 HG00673.paternal.f1_assembly_v2_genbank.fa.gz.idx ROI_seq.fa
./minimap2/minimap2 -x asm5 HG00735.maternal.f1_assembly_v2_genbank.fa.gz.idx ROI_seq.fa
./minimap2/minimap2 -x asm5 HG00735.paternal.f1_assembly_v2_genbank.fa.gz.idx ROI_seq.fa
./minimap2/minimap2 -x asm5 HG00741.maternal.f1_assembly_v2_genbank.fa.gz.idx ROI_seq.fa
./minimap2/minimap2 -x asm5 HG00741.paternal.f1_assembly_v2_genbank.fa.gz.idx ROI_seq.fa
./minimap2/minimap2 -x asm5 chm13.draft_v1.1.fasta.gz.idx ROI_seq.fa
EOF
```

prg-tk:
revision: 75fa20b41592941c9e6eef3f914d97788ee06b86
commands:

```
ls *.fa.gz > agc_inputs
\time -v ~/benchmark/pgr-tk/agc/agc create chm13.draft_v1.1.fasta.gz -i agc_inputs >
test.agc 2>> timing.log
echo test.agc > pgr_input
\time -v pgr-mdb pgr_input test 2>> timing.log
\time -v pgr-query test ROI_seq.fa pgr-query-out 2> pgr-query-out.log
```

Here is the list of the testing query sequences:

Regions of interest for testing querying					
Name	Reference	Chromosom	begin	end	Strand

MHC-C2	GRCh38	chr6	32313513	32992088	
RCCE	GRCh38	chr6	31976719	32117146	0
AMY	GRRh38	chr1	103542345	103798299	0
LPA	GRRh38	chr6	160529904	160666180	0
IGH	GRRh38	chr14	106205008	106874830	0
HLA-CB	GRRh38	chr6	31143427	31484914	0
ABO	GRRh38	chr9	133163441	133361030	0
TSPY1	GRRh38	chrY	9294496	9591276	0
15q15	GRRh38	chr15	43531685	43769928	0
16p21	GRRh38	chr16	28139916	28830868	0

Supplementary Table 2

We compared the query results for two selected regions and found them to be consistent. In these two cases, due to differences in their design, the "pgr-query" command only produced a single aligned region for each reference assembly, rather than multiple supplementary alignments. Similar to minimap2, "pgr-query" provides additional information about the hits, allowing the user to apply filters and define criteria to eliminate false positive alignments caused by repeats in more complex scenarios.

	pgr-query results		minimap2 results		consistent
MHC Class 2	begin	end	begin	end	
HG00438#1#JAHBCB010000040.1	23357242	24010477	23356506	24011428	Yes
HG00438#2#JAHBCA010000042.1	23362233	24141470	23361497	24142421	Yes
HG00621#1#JAHBCD010000020.1	23356282	24115439	23352369	24116390	Yes
HG00621#2#JAHBCC010000005.1	32265868	32906962	32264542	32910924	Yes
HG00673#1#JAHBBZ010000030.1	32179011	32823596	32177436	32824276	Yes
HG00673#2#JAHBBY010000031.1	886239	1474176	884899	1475258	Yes
HG00735#1#JAHBCH010000013.1	32366232	33038084	32364489	33042050	Yes
HG00735#2#JAHBCG010000038.1	3651996	4413560	3650989	4414240	Yes
HG00741#1#JAHALY010000025.1	23365046	24008887	23363908	24010574	Yes
HG00741#2#JAHALX010000077.1	25645945	26293199	25644213	26294150	Yes
chm13 chr6	32168394	32812380	32166819	32813462	Yes

AMY					
HG00438#1#JAHBCB010000015.1	33562835	33889251	33561142	33889429	Yes
			33657228	33801412	
			33761202	33865960	
HG00438#2#JAHBCA010000012.1	51600987	51760853	51599294	51761031	Yes
			51611167	51668018	
			51663126	51695258	
			51695374	51737568	
HG00621#1#JAHBCD010000034.1	2187959	2536123	2187725	2537760	Yes
			2211188	2315970	
			2253384	2410116	
			2469036	2509286	
HG00621#2#JAHBCC010000031.1	16758012	17011992	16756319	17012170	Yes
HG00673#1#JAHBBZ010000075.1	16748192	16908034	16746499	16908212	Yes
			16758372	16815202	
			16810310	16842663	
			16842559	16884745	
HG00673#1#JAHBBZ010000329.1	312	93447	22834	50859	
			22920	48155	
			22930	48307	
			24483	94099	
			8	87032	
HG00673#2#JAHBBY010000109.1	16763372	17111516	16761679	17111694	Yes
HG00735#1#JAHBCH010000004.1	101636258	101890244	101634565	101890422	Yes
HG00735#2#JAHBCG010000068.1	4817143	4977006	4815450	4977184	Yes
			4827323	4884173	
			4879281	4911562	
			4911529	4953720	
HG00741#1#JAHALY010000007.1	18847041	19219687	18846807	19221324	Yes

			18931609	19009465	
			19037277	19125244	
HG00741#2#JAHALX010000013.1	51662338	51916331	51660645	51916509	Yes
			51672517	51729368	
			51756716	51850848	
			51802317	51893042	
chm13:chr1	103392985	103835251	103391292	103835429	
			103680709	103699433	

Supplementary Table 3

In **Supplementary Table 3**, we compared the results of the pgr-query and minimap2 for a test set of pangenomic sequences. While pgr-query is designed to fetch homologous sequences from the database using long query sequences, rather than as a general sequence aligner, it is important to demonstrate its performance in fetching sequences accurately. We compared all hits larger than 10 kb from the pgr-query output for a set of 395 query sequences from the CMRG to the minimap2 output and found that the results are highly consistent in most cases. Some discrepancies are due to (1) short hits and (2) low minimap2 mapQV output. The pgr-query output might be more sensitive, such that some repetitive sequences are in the query output without proper filtering.

Supplementary Table 3a, we compare the unfiltered pgq-query output and the filtered output with hits that has more than 5 minimizer anchors found with the minimap2 output unfiltered or filtered by MapQV. Based on the hits from minimap2, pgr-query captures 97% to 99% hits, depending on the filtering criteria.

	Minimap2 (All)	Minimap2 (MapQV > 30)
minimap2 hits	3668	3190
overlapped pgr-query hits (filtered)	3601	3164
80% overlapped percentage	98.17%	99.18%
overlapped pgr-query hits (filtered)	3573	3164
80% overlapped percentage	97.41%	99.18%

Supplementary Table 3b, Base on the hit output from pgr-query, minimap2 capture 85% to 94% hits, depending on filtering criteria.

	unfiltered	filtered
pgr-query hits	3637	3589
overlapped minimap2 hits	3397	3396
80% overlapped percentage	93.40%	94.62%
overlapped minimap2 hits (MapQV>30)	3104	3103
80% overlapped percentage	85.35%	86.46%

Supplementary Table 3c, Pangenome Graph Construction Comparison. The measure resource usage for making index with different parameters.

Data: 97 haplotype human genome assembly							
w	k	r	Index file size (Gb)	elapse time (min:sec)	User Space CPU time (s)	System CPU time (s)	Memory Usage (Kbytes)
80	56	12	3	13:11.09	10749	455	35605852
80	56	8	6.1	14:21.31	10865	462	41329332
80	56	6	9.5	15:50.63	10966	472	48986112
80	56	4	15	18:26.03	11171	488	61270196
80	48	4	15	18:10.64	11159	485	59441552
80	32	4	15	17:58.06	11129	484	59576876
80	24	4	15	17:47.83	11139	482	59178764
64	56	4	17	19:13.54	11393	517	65174852
48	56	4	18	20:14.21	11685	543	70309960

command:

```
echo /wd/data/pgr-tk-HGRP-y1-evaluation-set-v0.agc > input

\time -v pgr-mdb -r 4  input  pgr-tk-HGRP-y1-evaluation-set-v0-r4 >& log_r4
\time -v pgr-mdb -r 6  input  pgr-tk-HGRP-y1-evaluation-set-v0-r6 >& log_r6
\time -v pgr-mdb -r 8  input  pgr-tk-HGRP-y1-evaluation-set-v0-r8 >& log_r8
```

```
\time -v pgr-mdb -r 12  input  pgr-tk-HGRP-y1-evaluation-set-v0-r12 >& log_r12

\time -v pgr-mdb -k 48  input  pgr-tk-HGRP-y1-evaluation-set-v0-k48 >& log_k48
\time -v pgr-mdb -k 32  input  pgr-tk-HGRP-y1-evaluation-set-v0-k32 >& log_k32
\time -v pgr-mdb -k 24  input  pgr-tk-HGRP-y1-evaluation-set-v0-k24 >& log_k24

\time -v pgr-mdb -w 64  input  pgr-tk-HGRP-y1-evaluation-set-v0-w64 >& log_w64
\time -v pgr-mdb -w 48  input  pgr-tk-HGRP-y1-evaluation-set-v0-w48 >& log_w48
```

Supplementary Table 4

Comparison of graph build time to seqwish and minigraph (input sequence data HLA Class II sequence from the 97 pangnome references)

Tool	Command Line	User time (seconds)	System time (seconds)	Elapsed (wall clock) time (min:sec)	memory usage (kb)
**seqwish**					
command	`wfmash HLA-ClassII_seq.fa HLA-ClassII_seq.fa -t 32 -X`	5270.32	4.39	3:09.82	878516
command	`seqwish -s HLA-ClassII_seq.fa -p HLA-ClassII_seq.paf -g HLA-ClassII_seq.gfa`	74.26	4.37	0:29.22	1619660
**minigraph**					
command	`minigraph -t 32 -cxggs chm13_HLA_C2.fa MHC*.fa > out.gfa`	799.91	41.2	13:19.61	2273760
**pgr-tk**					
command	`pgr-pbundle-decomp HLA-ClassII_seq.fa HLA-ClassII`	10.63	1.2	0:04.32	466448
command	`pgr-pbundle-decomp -r 3 HLA-ClassII_seq.fa HLA-ClassII_r3`	12.51	1.32	0:05.46	661628
command	`pgr-pbundle-decomp -r 1 HLA-ClassII_seq.fa HLA-ClassII_r1`	19.71	3.94	0:11.39	1294248

Tool	Command Line	number of vertices	number of edges	average vertex size (bp)	(Graph base length) / (total sequence length)
**seqwish**					
command	`wfmash HLA-ClassII_seq.fa HLA-ClassII_seq.fa -t 32 -X`				
command	`seqwish -s HLA-ClassII_seq.fa -p HLA-ClassII_seq.paf -g HLA-ClassII_seq.gfa`	121061	196640	122.4	0.1785
**minigraph**					
command	`minigraph -t 32 -cxggs chm13_HLA_C2.fa MHC*.fa > out.gfa`	293	409	3140.6	0.0111
**pgr-tk**					
command	`pgr-pbundle-decomp HLA-ClassII_seq.fa HLA-ClassII`	18258	29830	310.458	0.0683
command	`pgr-pbundle-decomp -r 3 HLA-ClassII_seq.fa HLA-ClassII_r3`	25274	40969	233.776	0.0712
command	`pgr-pbundle-decomp -r 1 HLA-ClassII_seq.fa HLA-ClassII_r1`	50773	80572	129.932	0.0795

Software versions used

seqwish:
source: https://github.com/ekg/seqwish.gi
revison:`f362f6f5ea89dbb6a0072a8b8ba215e663301d33`

minigraph
source: https://github.com/lh3/minigraph
revision: `3398263be225ba923140a1081b505b71f2cdf8fb`

pgr-pbundle-decomp (part of PGR-TK)
revision: `75fa20b41592941c9e6eef3f914d97788ee06b86`

The test sequence file "HLA-ClassII_seq.fa" comprises 147 sequences with an average length of 564,570 base pairs. It is important to note that not all sequences were incorporated in the Minigraph output as certain MHC Class II sequences displayed significant divergence from the CHM13 MHC Class II reference. The ratio of the total number of bases in the Minigraph output to the total number of bases in the input sequence file was observed to be significantly lower compared to the results produced by Seqwish and PGR-TK. The Seqwish graph was denser than the MAP-graph generated by `pgr-pbundle-decomp` and provided more detailed information that could be utilized for the direct identification of base-level differences.

On the other hand, PGR-TK demonstrated a significant advantage in terms of computational efficiency, with a construction time of the pangenome graphs that was 500x faster in terms of user CPU time and 60x faster in terms of wall clock time compared to Seqwish, and 75x faster and 160x faster, respectively, compared to Minigraph using default `pgr-pbundle-decomp` parameters.

Effect of the Parameter Choice to The MAP Vertex Sizes

Supplementary Figure 1

Vertex Sizes of the Chromosome 1 of Chm13 with different w and r, (min_span = 64).

Supplementary Figure 1a: the vertex size influences the resolution of the sequence being analyzed. Observing the vertex size distributions using various parameter sets, a flat region is observed followed by an exponential tail. To effectively query the database, it's best to ensure

that the query sequence length is a multiple of the average vertex size.

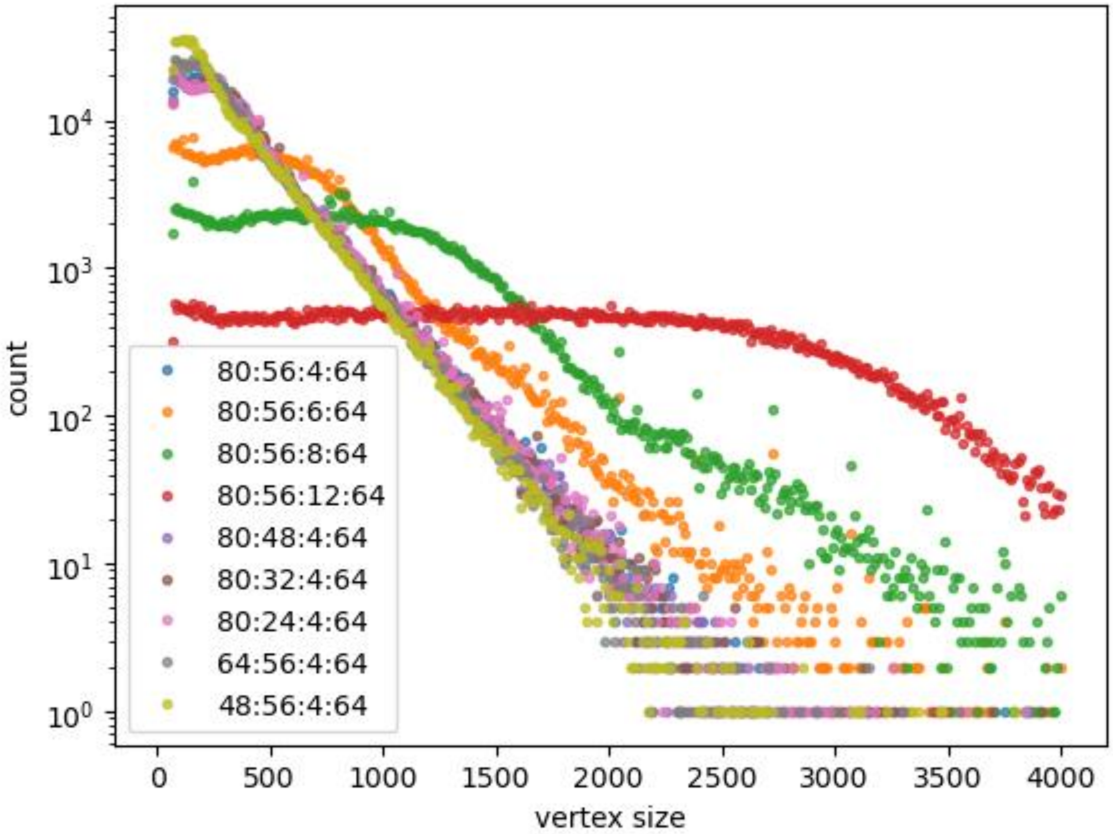

Supplementary Figure 1b: this vertex size distribution plot with the same parameter sets as those in **1a**, but with a small min_span. To reduce excessing minimizer in long simple repeat regions, we remove all pairs of minimizer anchors that are smaller than min_span to reduce unnecessary additional computation resources for processing simple repeat regions.

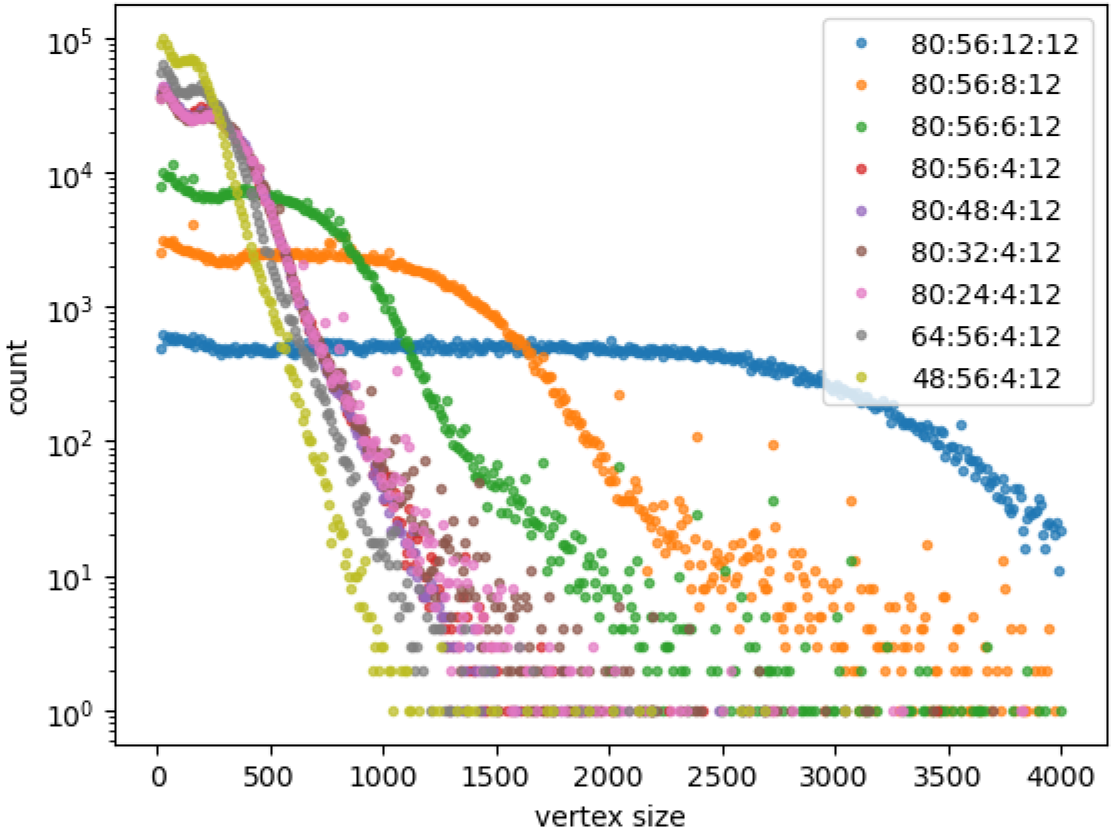

Supplementary Table 5

The descriptive statistics from the different set of parameter choice.

parameter set (w:k:r:m)	total vertex	media size	mean size	standard deviation	99.9%	99.0%
80:56:12:64	146967	1574	1690.1	1551.1	148956	24605
80:56:8:64	309494	754	802.6	519.4	7821	3388
80:56:6:64	484243	462	512.9	338.5	3551	2202
80:56:4:64	759890	269	326.9	235.0	2283	1600
80:48:4:64	756118	272	328.5	234.9	2248	1583
80:32:4:64	748297	275	331.9	237.4	2404	1620
80:24:4:64	742768	277	334.4	240.8	2349	1623
64:56:4:64	831991	236	298.5	221.4	2146	1515
48:56:4:64	903455	202	274.9	216.7	2124	1483

80:56:12:12	153192	1518	1621.4	1505.4	148956	23945
80:56:8:12	341088	695	728.2	484.5	7655	2841
80:56:6:12	583065	398	426.0	287.9	3003	1588
80:56:4:12	1159085	195	214.3	148.7	1204	822
80:48:4:12	1165335	196	213.1	148.5	1125	809
80:32:4:12	1132410	201	219.3	154.9	1620	930
80:24:4:12	1138924	200	218.1	154.2	1370	895
64:56:4:12	1402189	162	177.1	122.0	1000	688
48:56:4:12	1779213	128	139.6	95.4	777	558

Suggested Parameter Choice for Region of Size Up to 5Mb

Based on our observations in **Supplementary Table 4**, the parameter r has the greatest impact on the size of the vertices. To simplify the process, we recommend using the default values of w=48, k=56, and min_span=12 for general cases and adjusting the value of r based on the length of the sequence of interest. This approach can serve as a starting point and can be fine-tuned if specific detailed features are of interest.

Supplementary Table 6

w=48, k=56, min_span=12
r = floor(min(12, max(2, floor(2 * (mean(sequence lengths)/50000)^0.5))))

According to the formula, here is a table for the choice of r of different lengths of the sequences of interest:

sequence length(bp)	r
20,000	2
40,000	2
80,000	4
160,000	5
320,000	8

640,000	11
1,280,000	12
2,560,000	12
5,120,000	12

Generate MAP-Graph and Principal Bundle Decomposition for AMY and MHC regions

We use GRCh38 chr6:`32,313,513-32,992,088` (for MHC Class II) and GRCh38 chr1:`103,542,345-103,798,299` as the query sequences to find the homologous sequences in the pangenome reference database (`pgr-tk-HGRP-y1-evaluation-set-v0`):

```
cat << EOF  | tr " " "\t" > regions_interest
MHC-C2 hg38_tagged.fa chr6_hg38 32313513 32992088
AMY hg38_tagged.fa chr1_hg38 103542345 103798299 0
EOF
```

We use the `pgr-fetch-`seqs command in `PGR-TK` to get the two references:

```
pgr-fetch-seqs pgr-tk-HGRP-y1-evaluation-set-v0 \
-r regions_interest > ROI_seq.fa
```

Then, we use the `pgr-query` command to get the sequences in the pangenome reference. We merge the hits that are less than 100kb apart from each other:

```
pgr-query /wd/data/pgr-tk-HGRP-y1-evaluation-set-v0 \
/wd/results/pgr-out/ROI_seq.fa /wd/results/pgr-out/pg_seqs --merge-range-tol 100000
```

After fetching the sequence in the database, we filter out partial aligned contigs. With in the aligned contig, we generate the MAP-graph and the principal bundle decomposition by

```
pgr-pbundle-decomp -w ${w} -k ${k} -r ${r} \
        --min-span ${m} --bundle-length-cutoff 100 --min-branch-size 8 \
        ${fasta_file} /wd/results/pgr-out/${prefix}
```

For MHC class II, we choose w=48, k=56, r=7, m=12, and for AMY1A, we choose w=48, k=56, r=4, m=12 determined by the formula above.

The command pgr-pbundle-decomp generated the MAP-graph as gfa file and the principal bundle decomposition in bed format. For example, the first five bundles of Chm13 of the AMY1A region are represented as

```
chm13_tagged::chr1_chm13_103392985_103835251_0  174     27484   1:203:0:10:202:U
chm13_tagged::chr1_chm13_103392985_103835251_0  27428   27631   16:2:0:0:1:U
chm13_tagged::chr1_chm13_103392985_103835251_0  27575   50412   2:161:0:0:160:R
```

```
chm13_tagged::chr1_chm13_103392985_103835251_0  50356   51227   10:6:0:0:5:R
chm13_tagged::chr1_chm13_103392985_103835251_0  51171   55089   8:26:0:0:25:R
```

PGR-TK provides a command line tool for quick all pair-wise sparse alignment and compute distances between all pairwise sequences. With the distance we can perform hierarchical clustering to group bundles with similar structures for analysis or visualization. For example, the following command computes the distance based on the principal bundle decomposition

```
pgr-pbundle-bed2dist ${bed_file} ${prefix}
```

It generates three files:

```
${prefix}.dist # this file contains the distances between sequences

${prefix}.nwk  # the clustering tree in Newick format

${prefix}.ddg  # the file contains the dendrogram information for plotting a clustering
               # tree alone with the principal bundle decomposition with the command
               # pgr-pbundle-bed2svg
```

We can generate the principal decomposition plot with the command `pgr-pbundle-bed2svg`. For example, with the follow, we can generate a principal decomposition plot (`${prefix}.svg`) with the clustering dendrogram with annotation specified by a file ${prefix}.ord:

```
pgr-pbundle-bed2svg ${bed_file} ${prefix} \
              --track-range 250000 --track-tick-interval 10000
              --track-panel-width 1200 --stroke-width 1.2 \
              --annotations ${prefix}.ord \
              --ddg-file ${prefix}.ddg"
```

Please see more concrete examples in the git repo: https://github.com/GeneDx/pgr-tk

Comparing Principal Bundle Decomposition with Different Set of Parameters

We provide two illustrations of principal bundle decompositions, each with varying scales by changing the parameter sets. The first illustration, shown in Supplementary Figures 3a and 3b, pertains to a 130 kb region of interest containing the LPA KIV-II repeats. The second illustration, also shown in Supplementary Figures 3c and 3d, is of a 2.85 Mbp region located on chromosome 7 from positions 72752602 to 75600937 on GRCh38. This region is known to contain a microdeletion caused by nested repeats, which results in Williams-Beuren syndrome.

In **Supplementary Figure 2a**, there are 231 bundle segments spanning the 103,538 bp CHM13 LPA sequence, while only 93 bundle segments are present in **Supplementary Figure 2b**. The sparser decomposition with r=12 in **Supplementary Figure 2b** for this region may not provide sufficient detail for analyzing repeat elements in the sequences

In contrast, for the large 2.85 Mbp region, the choice r=12 provides a better representation of the overall structure (**Supplementary Figure 2c**), as it contains only 251 bundle segments out of the 2,916,749 bp chromosome 13 sequence, compared to the r=6 choice (**Supplementary Figure 2d**), which has 685 bundle segments. The higher number of bundle segments in the r=6 choice results in over-fragmentation of the sequences, making it more difficult to identify interesting repeats.

The `pgr-pbundle-decomp` command-line tool generates a summary of all contigs, providing valuable information for analysis by reporting the total number and average lengths of repetitive and non-repetitive fragments. This information can help the user make informed decisions if the default parameters suggested in **Supplementary Table 6** do not capture the desired features when comparing pangenome sequences.

Supplementary Figure 2

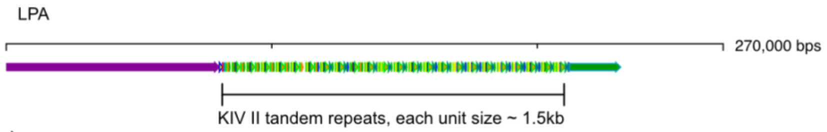

(a)

```
pgr-pbundle-decomp -w 48 -k 56 -r 4 --min-span 12 --bundle-length-cutoff 100
     --min-branch-size 8 pg_seqs.fa  LPA_pg_seqs. --min-cov 0
```

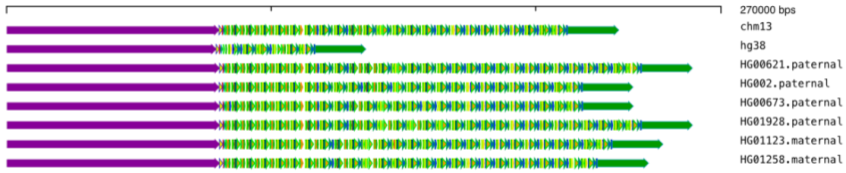

(b)

```
pgr-pbundle-decomp -w 48 -k 56 -r 12 --min-span 12 --bundle-length-cutoff 100
     --min-branch-size 8 pg_seqs.fa  LPA_pg_seqs. --min-cov 0
```

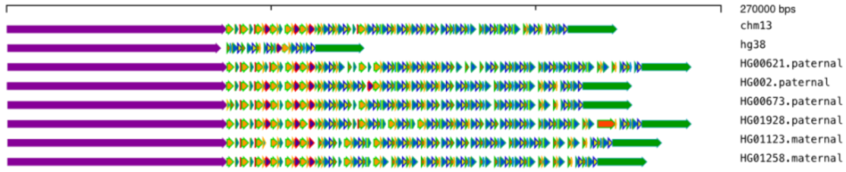

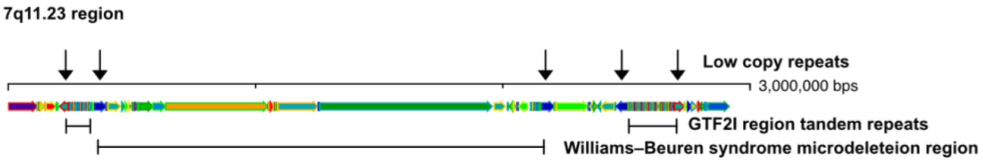

(c)

```
pgr-pbundle-decomp -w 48 -k 56 -r 12 --min-span 12 --bundle-length-cutoff 100
     --min-branch-size 8 pg_seqs.fa  WBS_pg_seqs. --min-cov 0
```

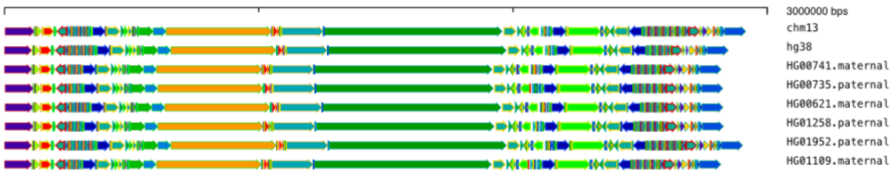

(d)

```
pgr-pbundle-decomp -w 48 -k 56 -r 6 --min-span 12 --bundle-length-cutoff 100
     --min-branch-size 8 pg_seqs.fa  WBS_pg_seqs. --min-cov 0
```

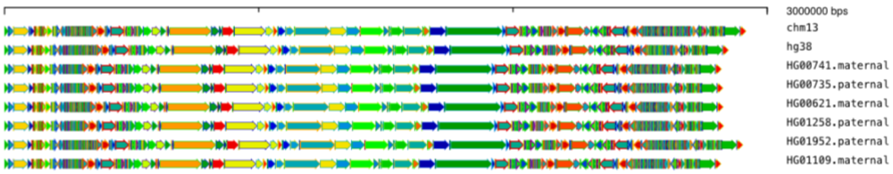

AMY1A repeat dot plots and principal bundle decomposition plots

Supplementary Figure 3

Supplementary Figure 3a

AMY1A repeat dot plots and principal bundle decomposition plots

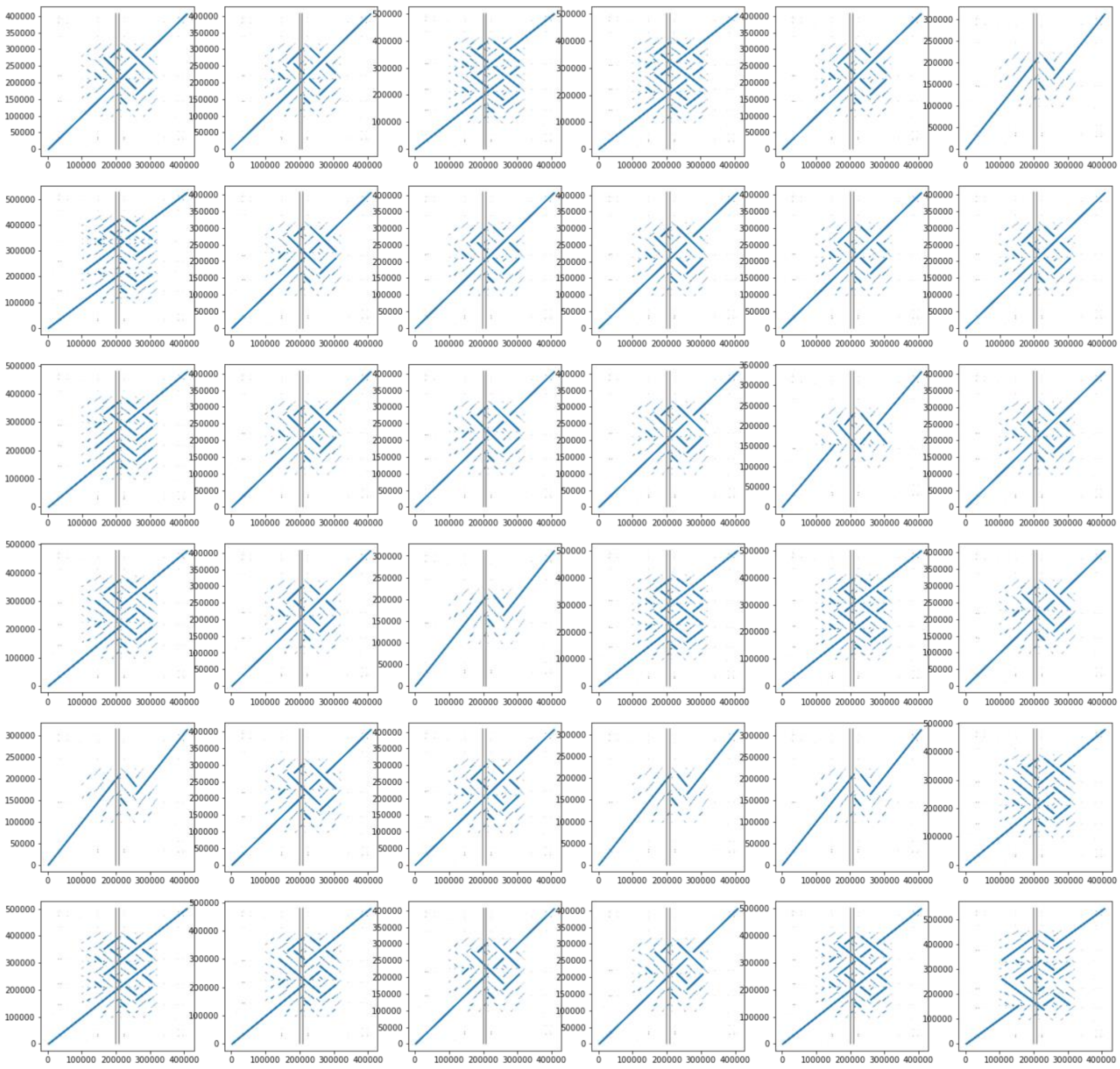

Supplementary Figure 3b: The principal bundle plots of the AMY1A repeat regions. The black short bars indicate the regions homologous to AMY1A sequence.

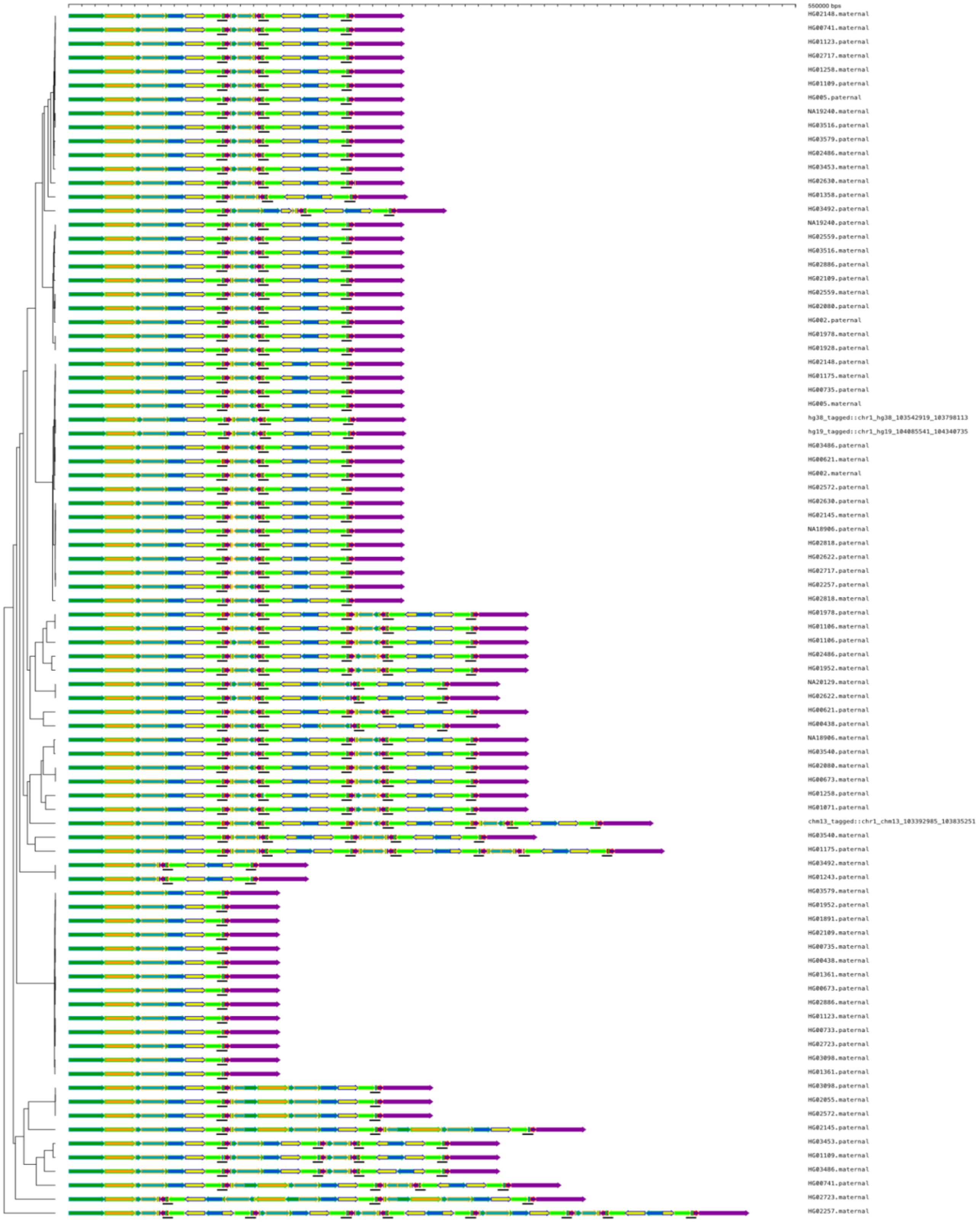

550000 bps
HG02148.maternal
HG00741.maternal
HG01123.paternal
HG02717.maternal
HG01258.maternal
HG01109.paternal
HG005.paternal
NA19240.maternal
HG03516.paternal
HG03579.paternal
HG02486.maternal
HG03453.maternal
HG02630.maternal
HG01358.paternal
HG03492.paternal
NA19240.paternal
HG02559.paternal
HG03516.maternal
HG02886.paternal
HG02109.paternal
HG02559.maternal
HG02080.paternal
HG002.paternal
HG01978.maternal
HG01928.paternal
HG02148.paternal
HG01175.maternal
HG00735.paternal
HG005.maternal
hg38_tagged::chr1_hg38_103542919_103798113
hg19_tagged::chr1_hg19_104085541_104340735
HG03486.paternal
HG00621.maternal
HG002.maternal
HG02572.paternal
HG02630.paternal
HG02145.maternal
NA18906.paternal
HG02818.paternal
HG02622.paternal
HG02717.paternal
HG02257.paternal
HG02818.maternal
HG01978.paternal
HG01106.maternal
HG01106.paternal
HG02486.paternal
HG01952.maternal
NA20129.maternal
HG02622.maternal
HG00621.paternal
HG00438.paternal
NA18906.maternal
HG03540.paternal
HG02080.maternal
HG00673.maternal
HG01258.paternal
HG01071.paternal
chm13_tagged::chr1_chm13_103392985_103835251
HG03540.maternal
HG01175.paternal
HG03492.maternal
HG01243.paternal
HG03579.maternal
HG01952.paternal
HG01891.paternal
HG02109.maternal
HG00735.maternal
HG00438.maternal
HG01361.maternal
HG00673.paternal
HG02886.maternal
HG01123.maternal
HG00733.paternal
HG02723.paternal
HG03098.maternal
HG01361.paternal
HG03098.paternal
HG02055.maternal
HG02572.maternal
HG02145.paternal
HG03453.paternal
HG01109.maternal
HG03486.maternal
HG00741.paternal
HG02723.maternal
HG02257.maternal

GIAB CMRG cases

Supplementary Figure 4

(a) LMF1

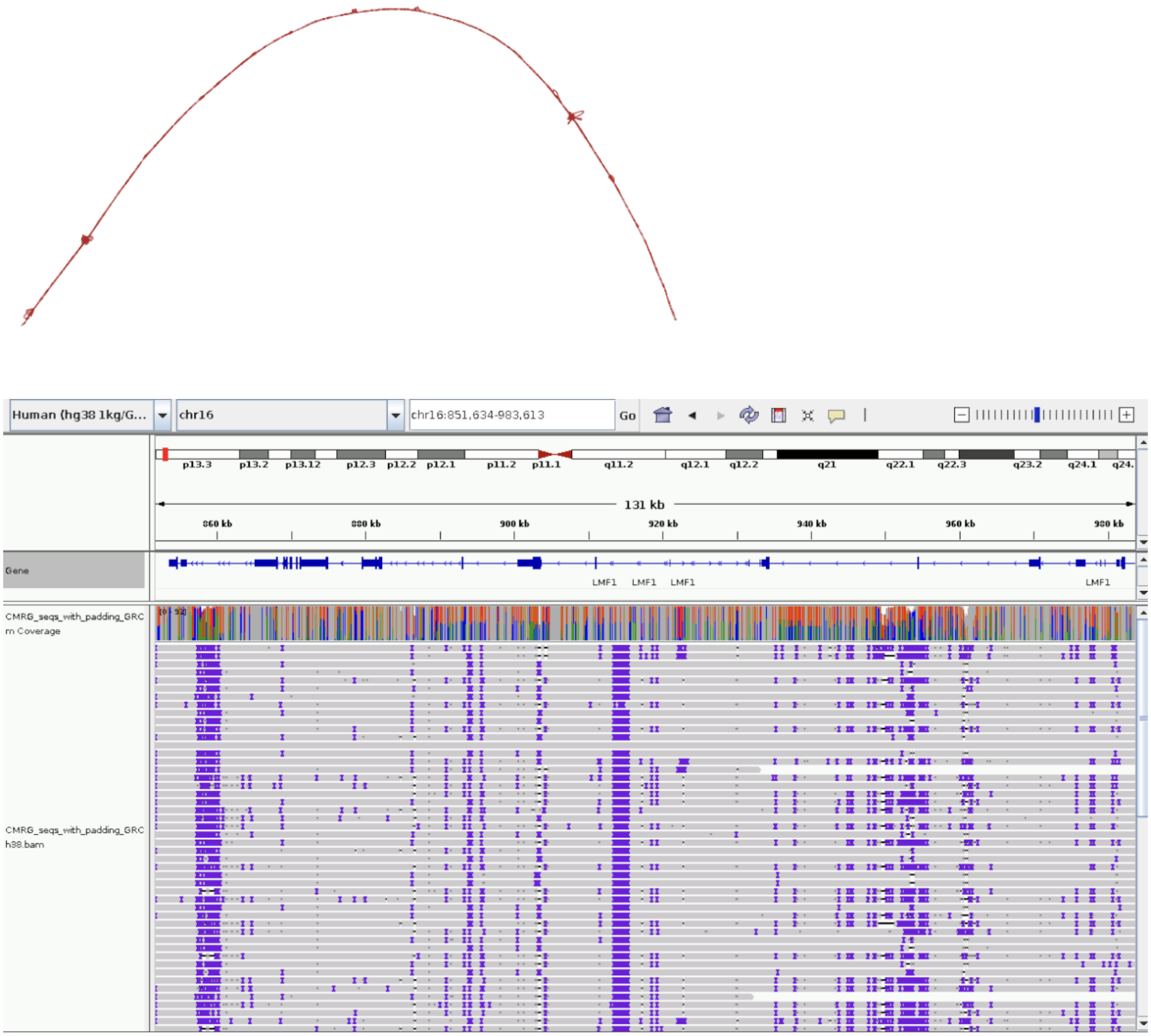

(b) Comparing the PAV structural variant calls indicated by the black auxiliary tracks to the principal bundle decomposition illustrates how the SV calls correspond to the changes in the principal bundles between each individual genome and the reference used for the SV call

(GRCh38, the top track).

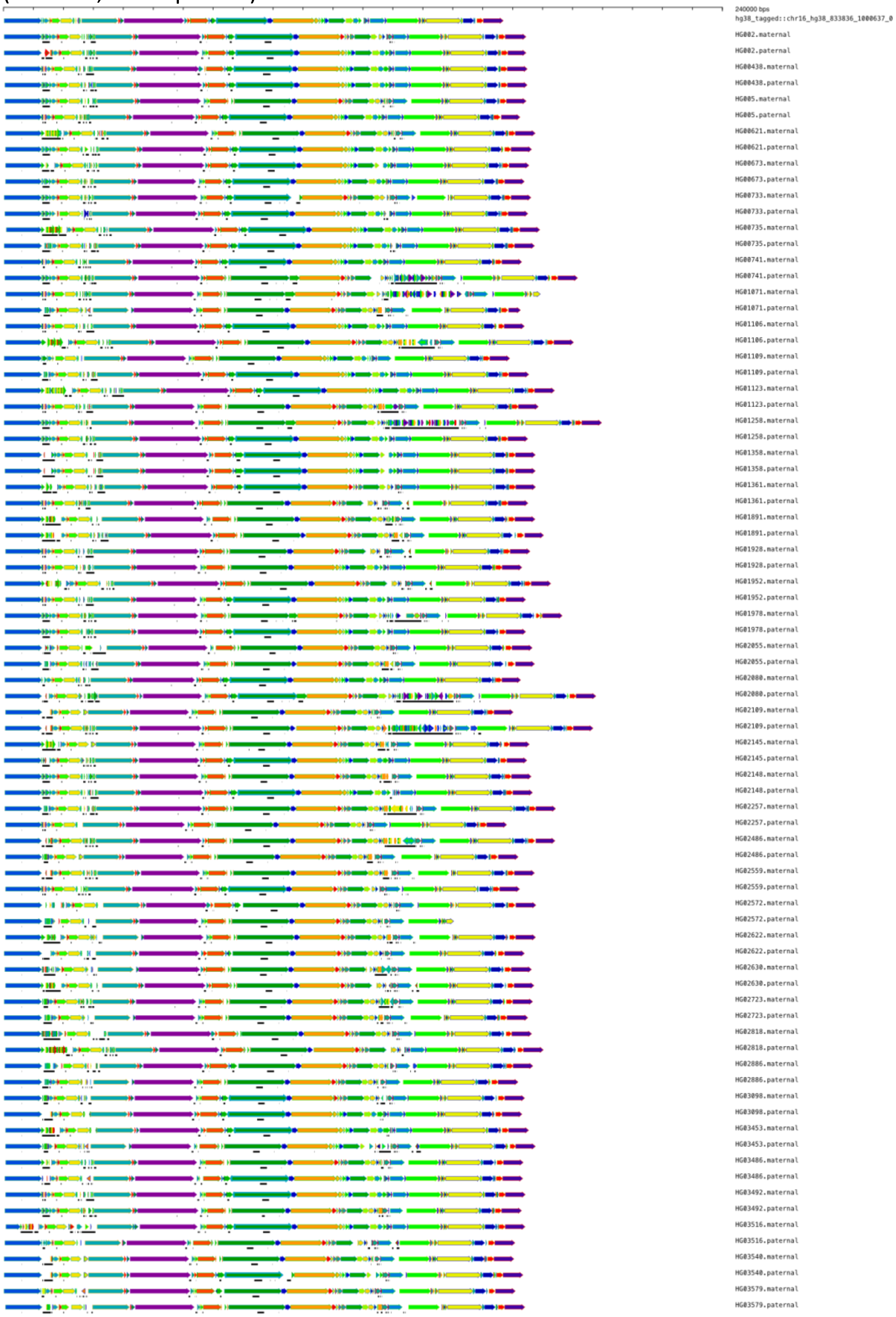

(b) ANKRD11

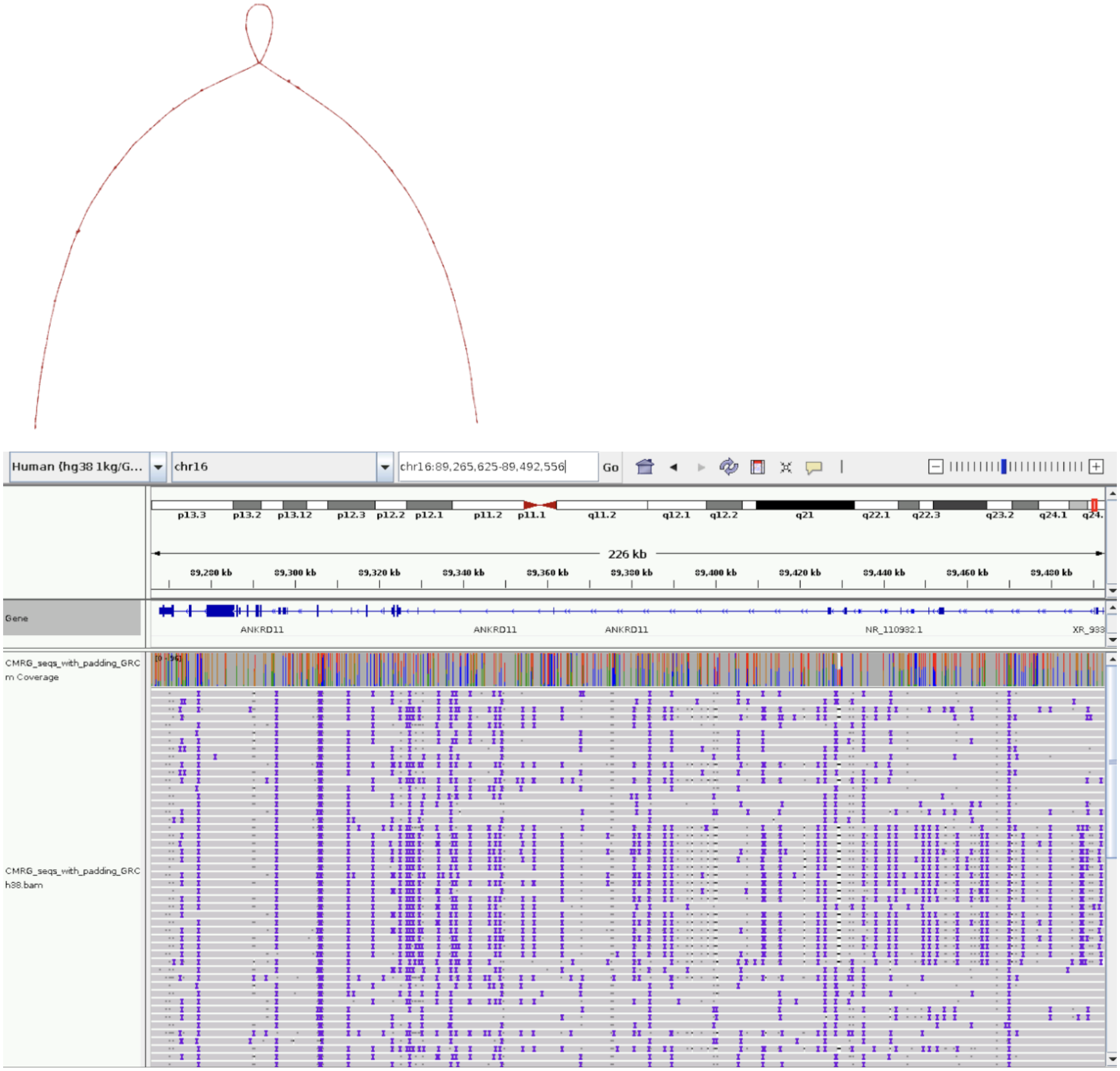
Human (hg38 1kg/G...
chr16
chr16:89,265,625-89,492,556
Go
p13.3
p13.2
p13.12
p12.3
p12.2
p12.1
p11.2
p11.1
q11.2
q12.1
q12.2
q21
q22.1
q22.3
q23.2
q24.1
226 kb
89,280 kb
89,300 kb
89,320 kb
89,340 kb
89,360 kb
89,380 kb
89,400 kb
89,420 kb
89,440 kb
89,460 kb
89,480 kb
Gene
ANKRD11
ANKRD11
ANKRD11
NR_110932.1
CMRG_seqs_with_padding_GRCh38.bam Coverage
CMRG_seqs_with_padding_GRCh38.bam

(c) SRGAP2

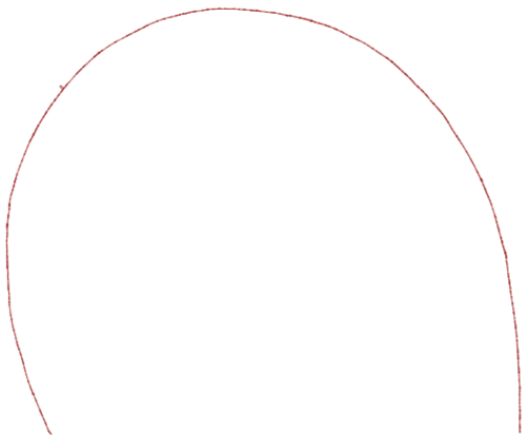

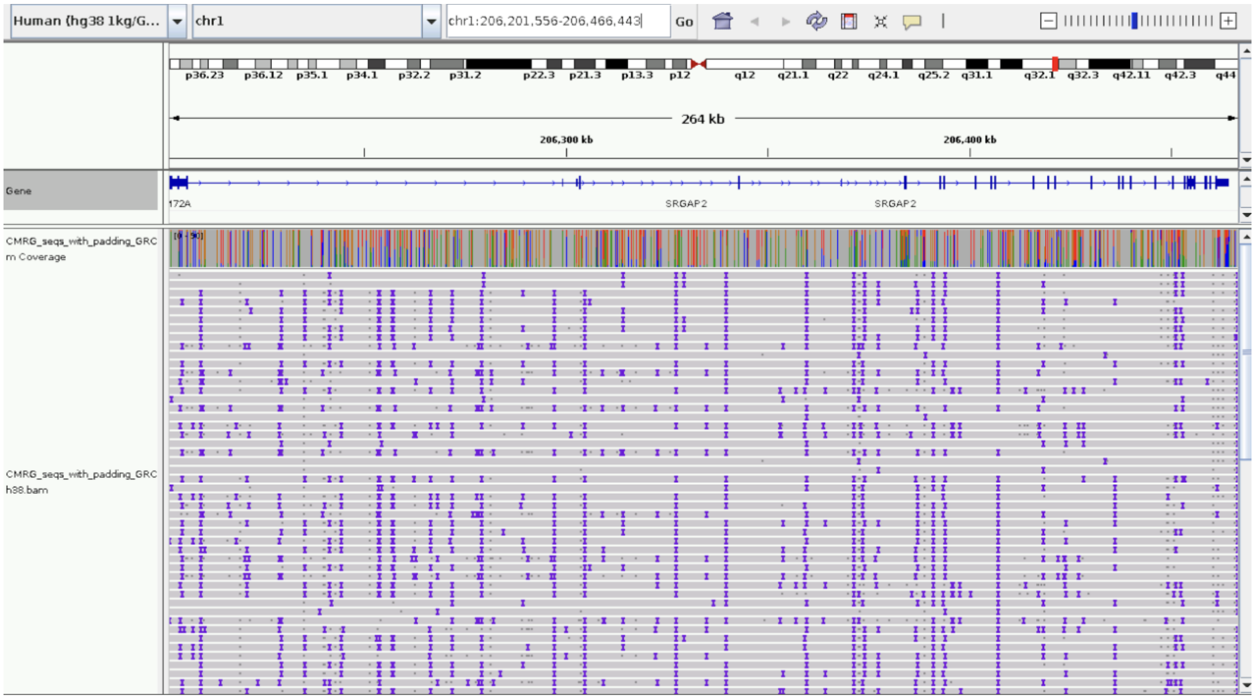
Human (hg38 1kg/G...
chr1
chr1:206,201,556-206,466,443
Go
p36.23
p36.12
p35.1
p34.1
p32.2
p31.2
p22.3
p21.3
p13.3
p12
q12
q21.1
q22
q24.1
q25.2
q31.1
q32.1
q32.3
q42.11
q42.3
q44
264 kb
206,300 kb
206,400 kb
Gene
172A
SRGAP2
SRGAP2
CMRG_seqs_with_padding_GRCh38.bam Coverage
CMRG_seqs_with_padding_GRCh38.bam

(d) KMT2C

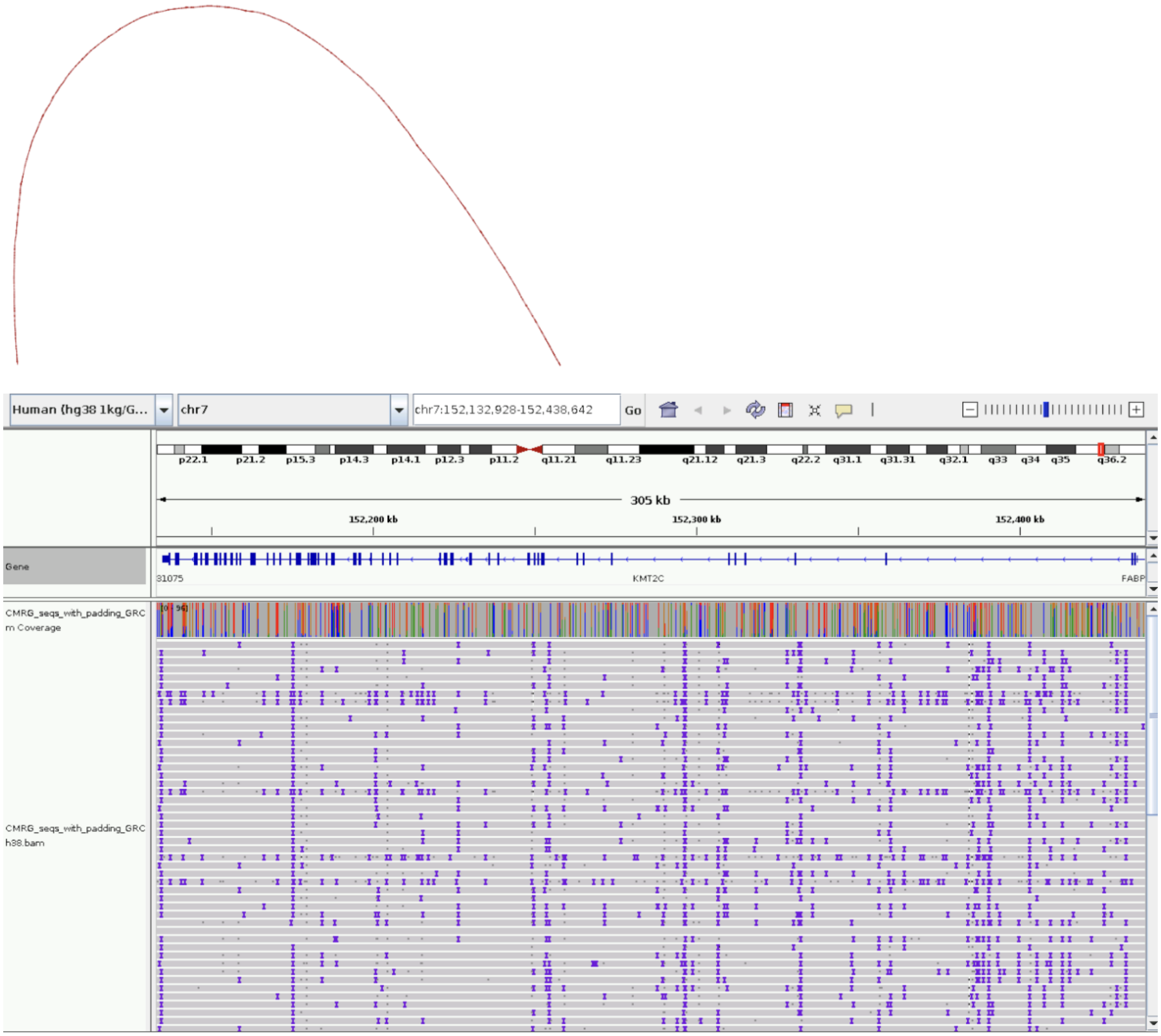

Human (hg38 1kg/G...
chr7
chr7:152,132,928-152,438,642
Go
p22.1
p21.2
p15.3
p14.3
p14.1
p12.3
p11.2
q11.21
q11.23
q21.12
q21.3
q22.2
q31.1
q31.31
q32.1
q33
q34
q35
q36.2
305 kb
152,200 kb
152,300 kb
152,400 kb
Gene
KMT2C
CMRG_seqs_with_padding_GRCm Coverage
CMRG_seqs_with_padding_GRCh38.bam

(e) LPA

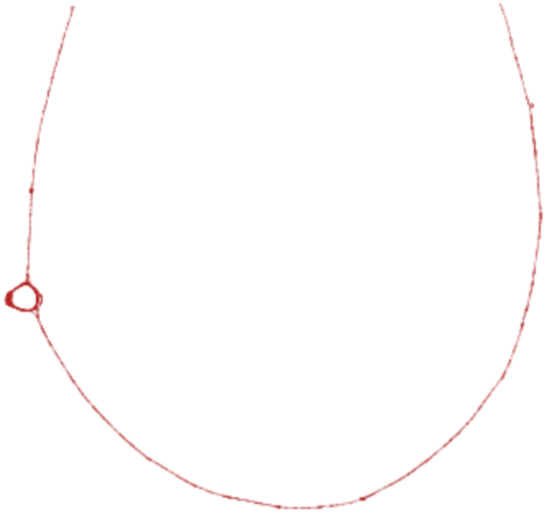

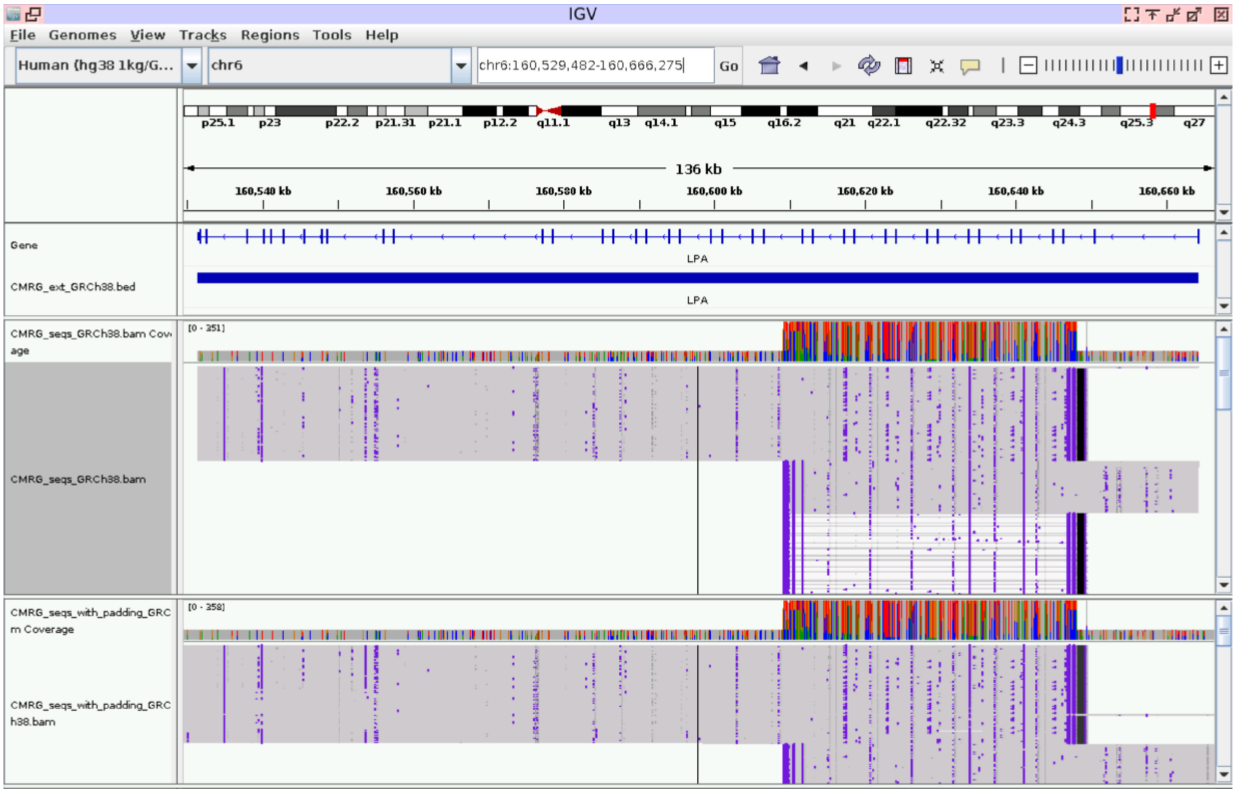
IGV
File Genomes View Tracks Regions Tools Help
Human (hg38 1kg/G...
chr6
chr6:160,529,482-160,666,275
Go
p25.1 p23 p22.2 p21.31 p21.1 p12.2 q11.1 q13 q14.1 q15 q16.2 q21 q22.1 q22.32 q23.3 q24.3 q25.3 q27
136 kb
160,540 kb 160,560 kb 160,580 kb 160,600 kb 160,620 kb 160,640 kb 160,660 kb
Gene
LPA
CMRG_ext_GRCh38.bed
LPA
CMRG_seqs_GRCh38.bam Coverage
[0 - 351]
CMRG_seqs_GRCh38.bam
CMRG_seqs_with_padding_GRCh38.bam Coverage
[0 - 358]
CMRG_seqs_with_padding_GRCh38.bam

(f) MUC4

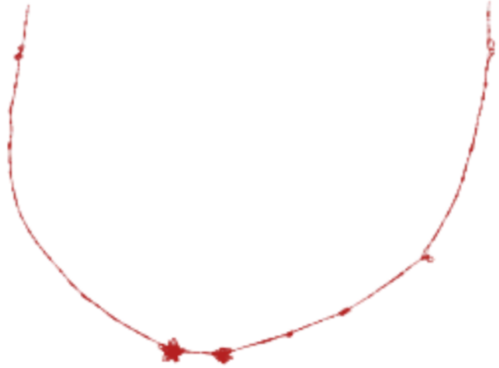

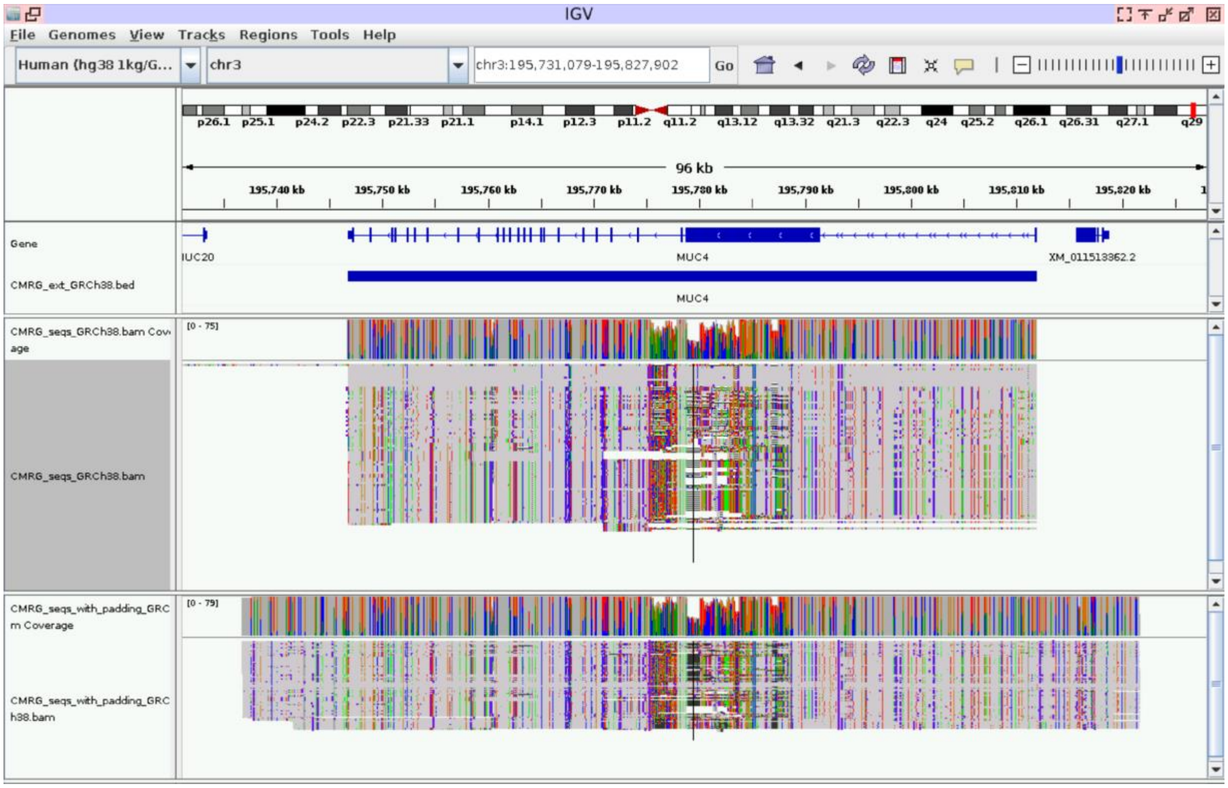

IGV
File Genomes View Tracks Regions Tools Help
Human (hg38 1kg/G...
chr3
chr3:195,731,079-195,827,902
Go
p26.1 p25.1 p24.2 p22.3 p21.33 p21.1 p14.1 p12.3 p11.2 q11.2 q13.12 q13.32 q21.3 q22.3 q24 q25.2 q26.1 q26.31 q27.1 q29
96 kb
195,740 kb 195,750 kb 195,760 kb 195,770 kb 195,780 kb 195,790 kb 195,800 kb 195,810 kb 195,820 kb
Gene
CMRG_ext_GRCh38.bed
MUC4
XM_011513362.2
CMRG_seqs_GRCh38.bam Coverage
[0 - 75]
CMRG_seqs_GRCh38.bam
CMRG_seqs_with_padding_GRCh38.bam Coverage
[0 - 79]
CMRG_seqs_with_padding_GRCh38.bam

(g) MUC3A

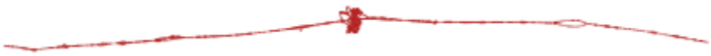

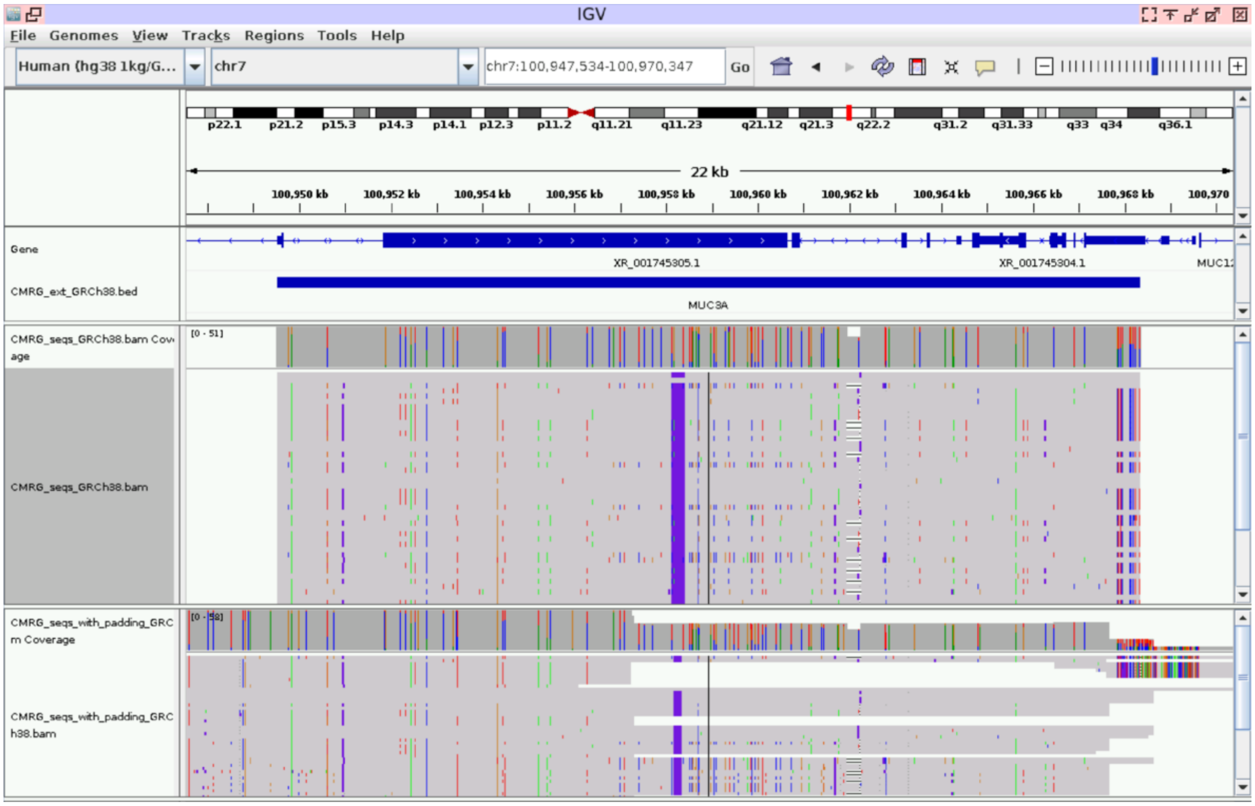
IGV
File Genomes View Tracks Regions Tools Help
Human (hg38 1kg/G...
chr7
chr7:100,947,534-100,970,347
Go
p22.1 p21.2 p15.3 p14.3 p14.1 p12.3 p11.2 q11.21 q11.23 q21.12 q21.3 q22.2 q31.2 q31.33 q33 q34 q36.1
22 kb
100,950 kb 100,952 kb 100,954 kb 100,956 kb 100,958 kb 100,960 kb 100,962 kb 100,964 kb 100,966 kb 100,968 kb 100,970
Gene
CMRG_ext_GRCh38.bed
XR_001745805.1
XR_001745804.1
MUC3A
CMRG_seqs_GRCh38.bam Coverage
[0 - 51]
CMRG_seqs_GRCh38.bam
CMRG_seqs_with_padding_GRCm Coverage
CMRG_seqs_with_padding_GRCh38.bam

(h) KATNAL2

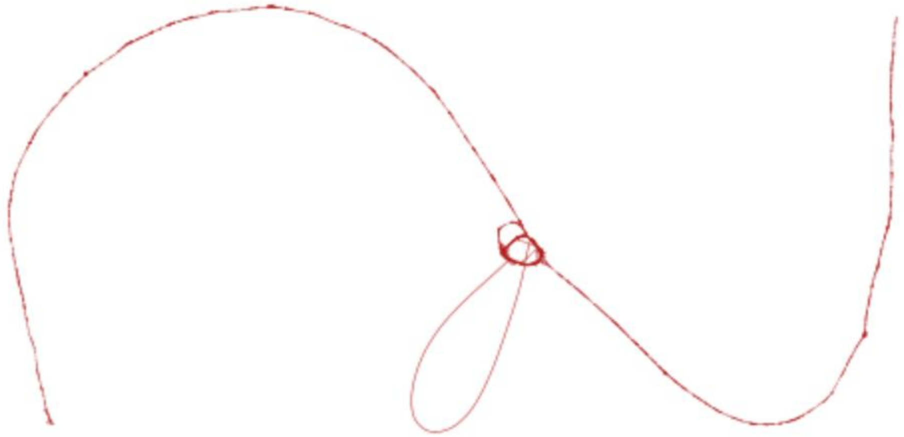

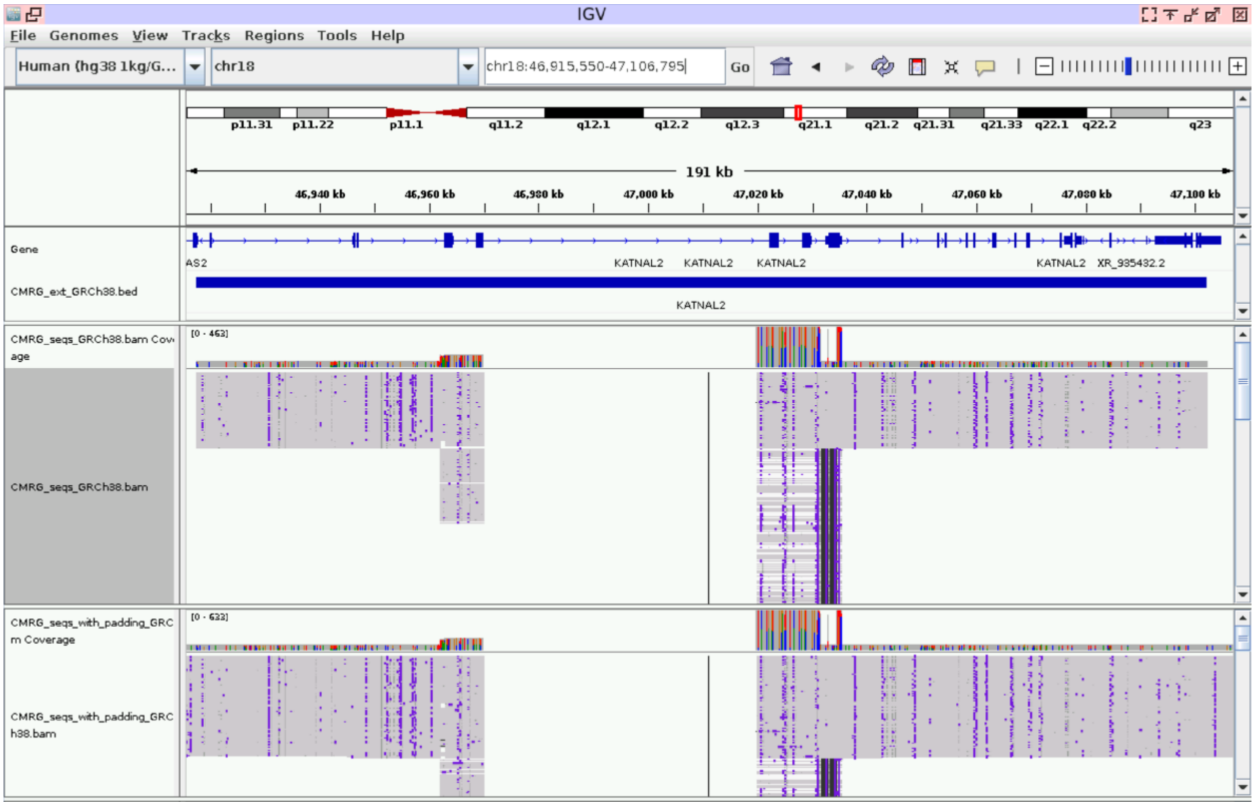

IGV
File Genomes View Tracks Regions Tools Help
Human (hg38 1kg/G...
chr18
chr18:46,915,550-47,106,795
Go
p11.31 p11.22 p11.1 q11.2 q12.1 q12.2 q12.3 q21.1 q21.2 q21.31 q21.33 q22.1 q22.2 q23
191 kb
46,940 kb 46,960 kb 46,980 kb 47,000 kb 47,020 kb 47,040 kb 47,060 kb 47,080 kb 47,100 kb
Gene
AS2
KATNAL2 KATNAL2 KATNAL2 KATNAL2 XR_935432.2
CMRG_ext_GRCh38.bed
KATNAL2
CMRG_seqs_GRCh38.bam Coverage
[0 - 463]
CMRG_seqs_GRCh38.bam
CMRG_seqs_with_padding_GRCh38.bam Coverage
[0 - 633]
CMRG_seqs_with_padding_GRCh38.bam

(i) FLG

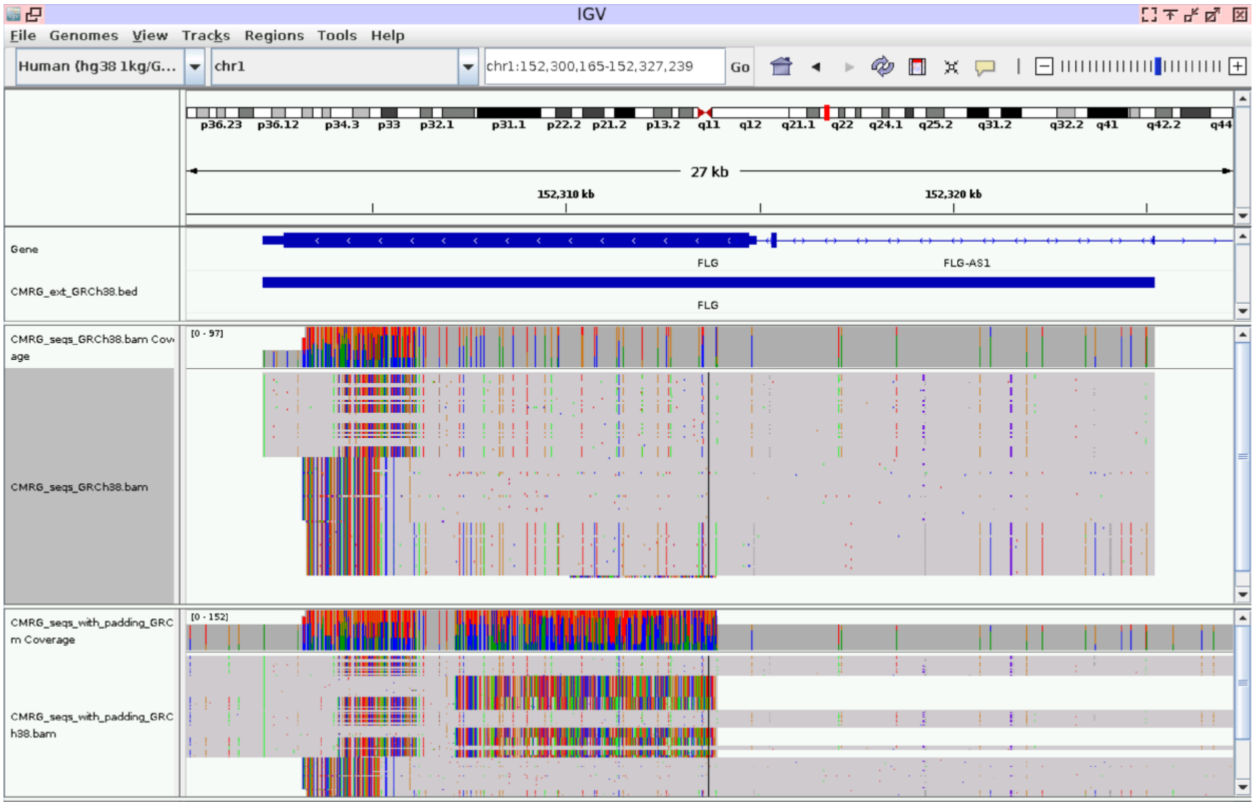
IGV
File Genomes View Tracks Regions Tools Help
Human (hg38 1kg/G...
chr1
chr1:152,300,165-152,327,239
Go
p36.23 p36.12 p34.3 p33 p32.1 p31.1 p22.2 p21.2 p13.2 q11 q12 q21.1 q22 q24.1 q25.2 q31.2 q32.2 q41 q42.2 q44
27 kb
152,310 kb
152,320 kb
Gene
CMRG_ext_GRCh38.bed
FLG
FLG-AS1
FLG
CMRG_seqs_GRCh38.bam Coverage
[0 - 97]
CMRG_seqs_GRCh38.bam
CMRG_seqs_with_padding_GRCm Coverage
[0 - 152]
CMRG_seqs_with_padding_GRCh38.bam

SNTG2 and KMT2

Supplementary Figure 5

Supplementary Figure 5a

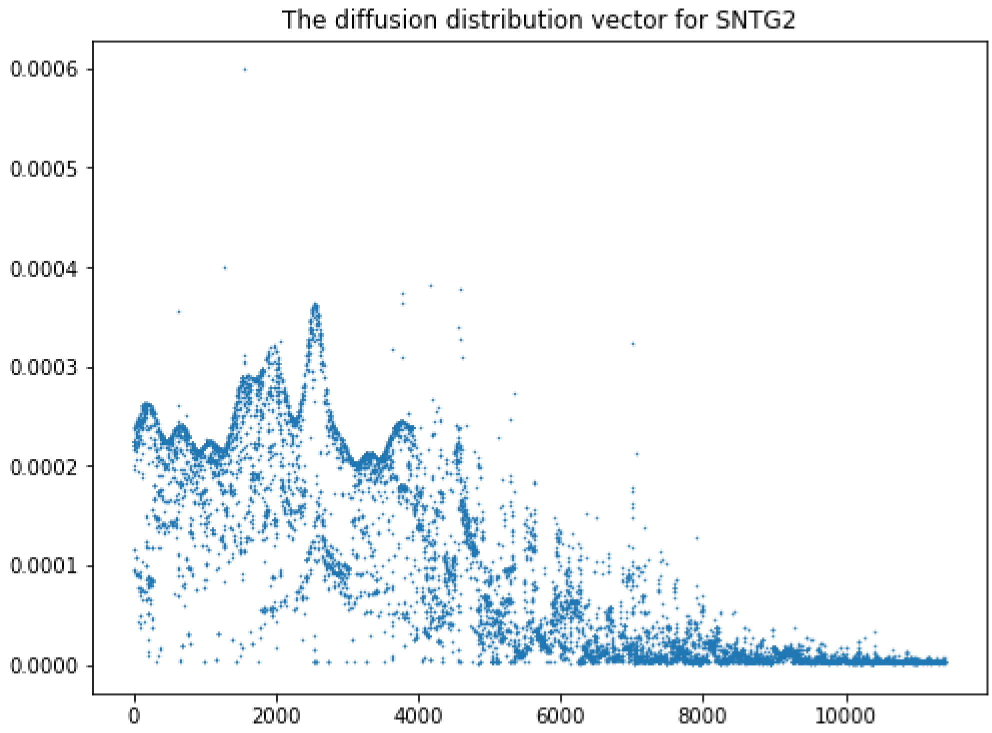

Supplementary Figure 5b: PCA plot for SNTG2 (Highest Entropy in the CMRG gene set)

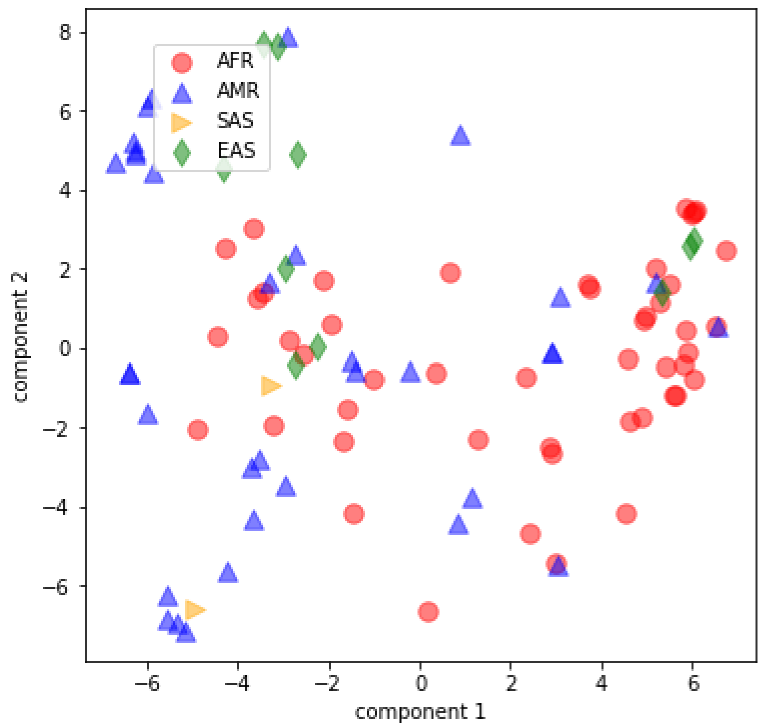

Supplementary Figure 5c: PCA plot for KMT2C (Highest Entropy in the CMRG gene set)

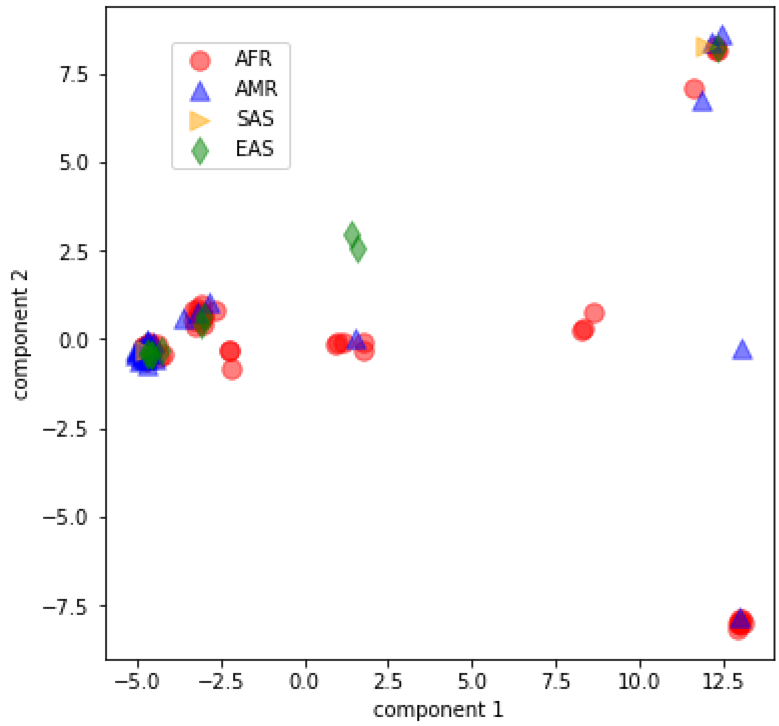

The distribution of the vertex weight on the principal bundle vertices for MHC class II, *AMY1A* and *LPA*

Supplementary Figure 6

The distribution of the vertex weight on the principal bundle vertices and non-principal bundle vertices for the three cases MHC class II, AMY1A and LPA regions.

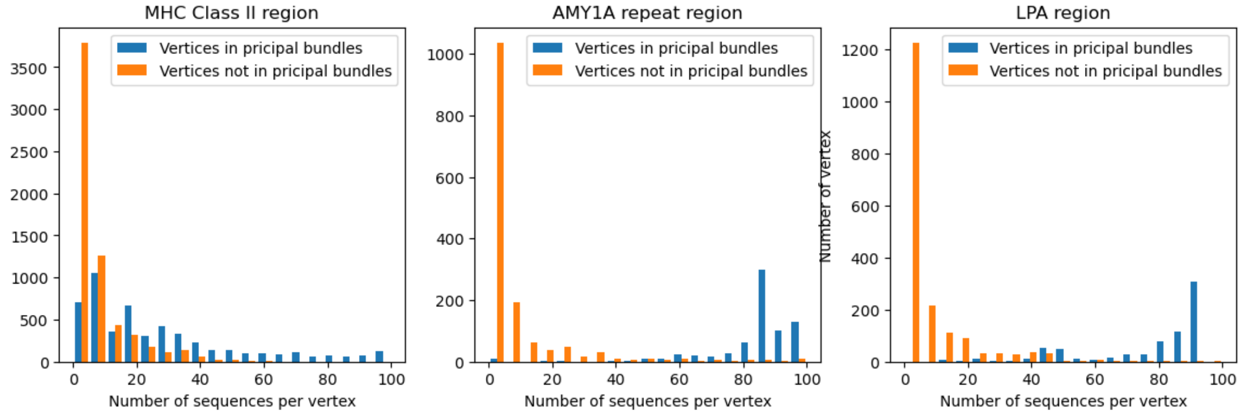

LPA, KIV-II repeats principal bundle decomposition plot

Supplementary Figure 7: LPA, KIV-II repeats principal bundle decomposition plot

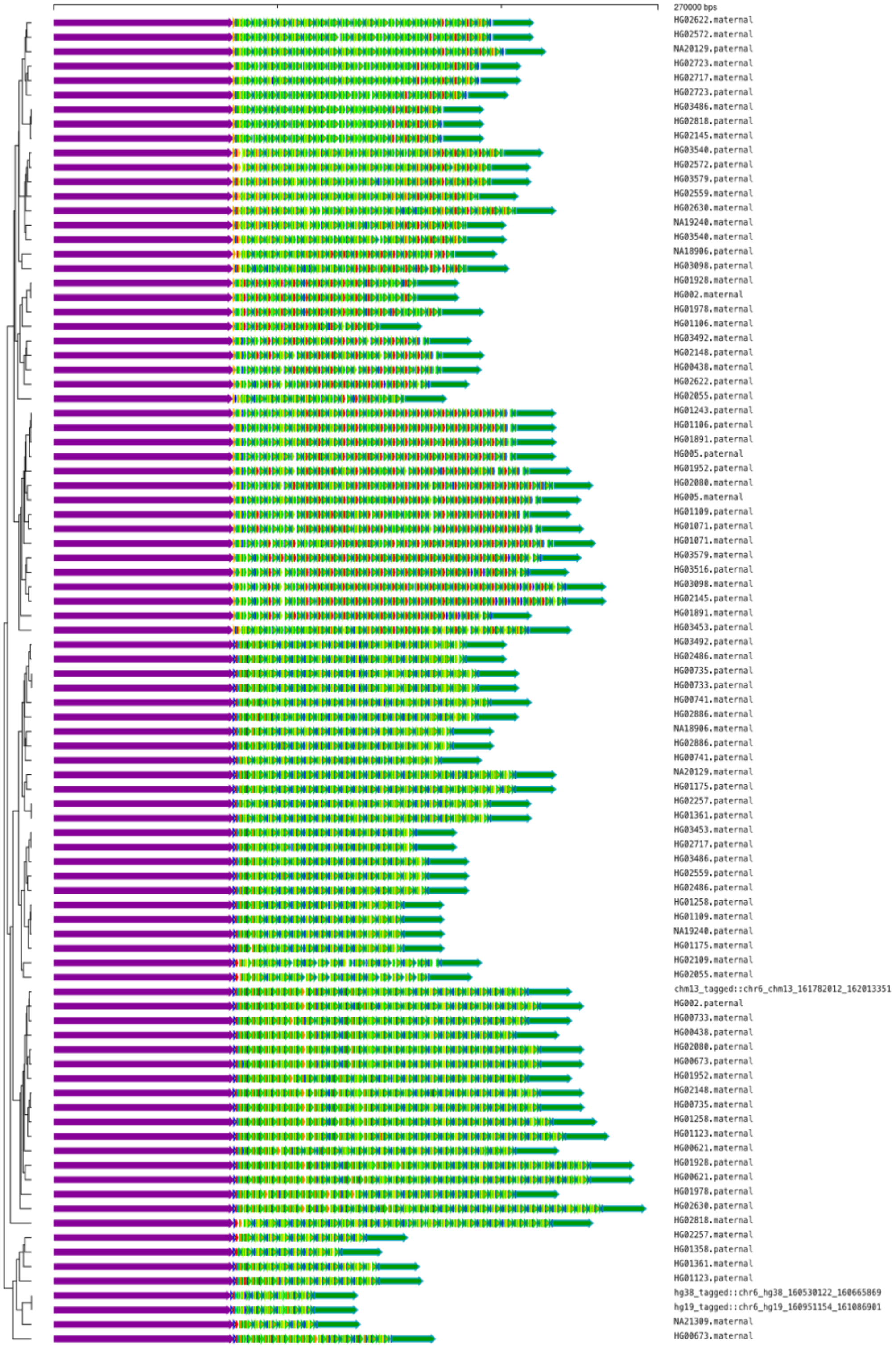

Principal bundle plot for KATNAL2

Supplementary Figure 8

Principal bundle plot for KATNAL2: GRCh38 chr18:46905550-47116795 showing different numbers of the repeat.

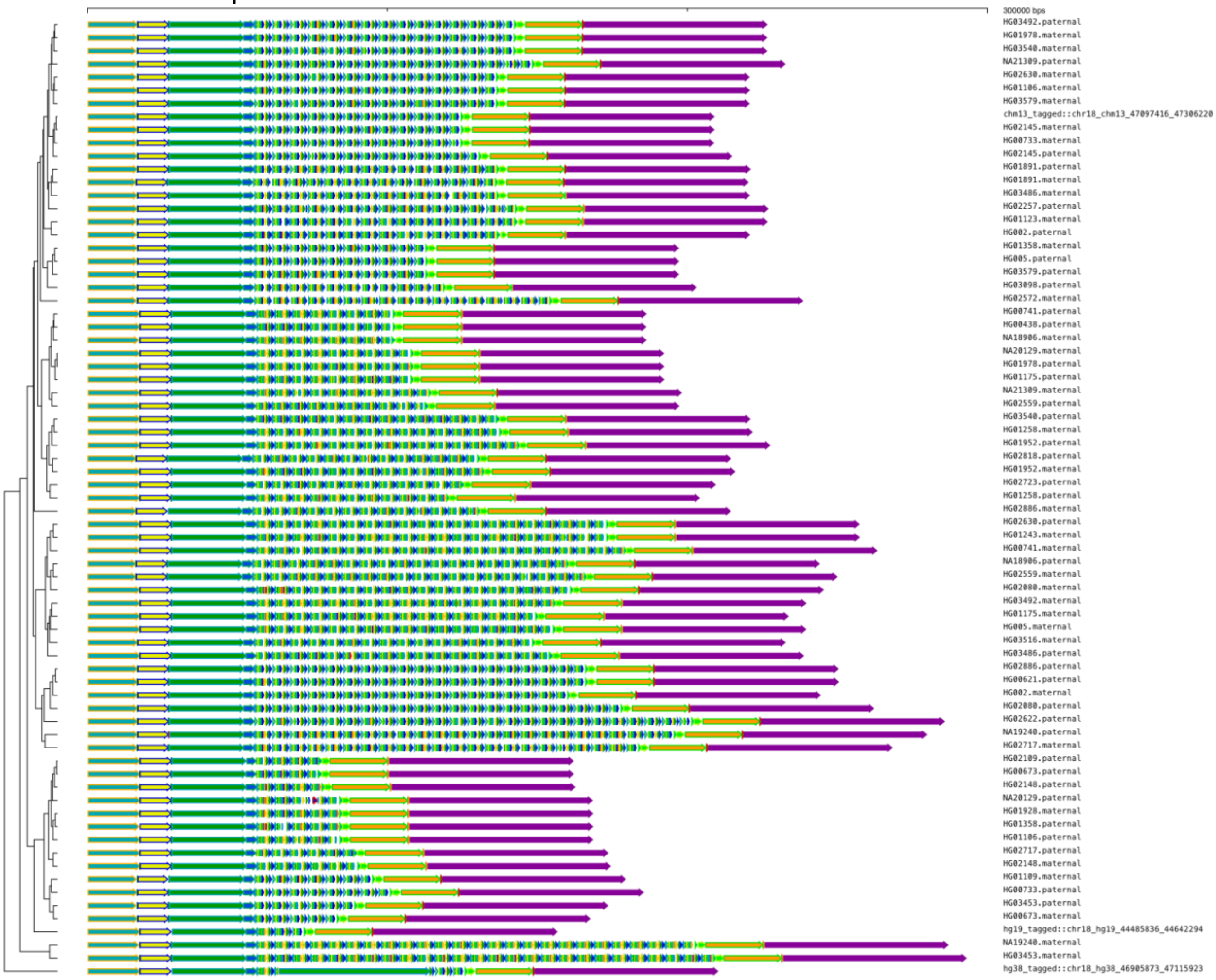

Supplement: Supplementary file 1 — Supplementary Tables 1–6 and Figs. 1–8. [file 41592_2023_1914_MOESM1_ESM.pdf]
