# Supplementary material for: Multiscale analysis of pangenomes enables improved representation of genomic diversity for repetitive and clinically relevant genes
Source: Nat Methods. 2023 Jun 26;20(8):1213–21. doi: 10.1038/s41592-023-01914-y (PMC10406601; doi:10.1038/s41592-023-01914-y)
Supplement: Supplementary file 2 — Reporting Summary [file 41592_2023_1914_MOESM2_ESM.pdf]

nature research

Corresponding author(s): CHEN-SHAN CHIN

Last updated by author(s): Apr 10, 2023

Reporting Summary

Nature Research wishes to improve the reproducibility of the work that we publish. This form provides structure for consistency and transparency in reporting. For further information on Nature Research policies, see our Editorial Policies and the Editorial Policy Checklist.

Statistics

For all statistical analyses, confirm that the following items are present in the figure legend, table legend, main text, or Methods section.

n/a	Confirmed	
☒	☐	The exact sample size (*n*) for each experimental group/condition, given as a discrete number and unit of measurement
☒	☐	A statement on whether measurements were taken from distinct samples or whether the same sample was measured repeatedly
☒	☐	The statistical test(s) used AND whether they are one- or two-sided<br>*Only common tests should be described solely by name; describe more complex techniques in the Methods section.*
☒	☐	A description of all covariates tested
☒	☐	A description of any assumptions or corrections, such as tests of normality and adjustment for multiple comparisons
☐	☒	A full description of the statistical parameters including central tendency (e.g. means) or other basic estimates (e.g. regression coefficient) AND variation (e.g. standard deviation) or associated estimates of uncertainty (e.g. confidence intervals)
☒	☐	For null hypothesis testing, the test statistic (e.g. *F*, *t*, *r*) with confidence intervals, effect sizes, degrees of freedom and *P* value noted<br>*Give P values as exact values whenever suitable.*
☒	☐	For Bayesian analysis, information on the choice of priors and Markov chain Monte Carlo settings
☒	☐	For hierarchical and complex designs, identification of the appropriate level for tests and full reporting of outcomes
☒	☐	Estimates of effect sizes (e.g. Cohen's *d*, Pearson's *r*), indicating how they were calculated

Our web collection on statistics for biologists contains articles on many of the points above.

Software and code

Policy information about availability of computer code

Data collection

The scripts to processes the origin data are in https://github.com/GeneDX/pgr-tk-notebooks/tree/main/pgr-tk-sequence-source (link included in the manuscript).

software version:
AGC (Assembled Genomes Compressor) v. 2.0 [build 20220405.1]
PGR-TK: revision: 75fa20b41592941c9e6eef3f914d97788ee06b86 and after

Data analysis

The following third party software are used in analysis

minimap2:
source: https://github.com/lh3/minimap2
revision: 01b98e8e52a8acfed5a9d57853f028267eaf045f

seqwish:
source: https://github.com/ekg/seqwish.gi
revison:f362f6f5ea89dbb6a0072a8b8ba215e663301d33

minigraph
source: https://github.com/lh3/minigraph
revision: 3398263be225ba923140a1081b505b71f2cdf8fb

rustc: 1.68.2 (9eb3afe9e 2023-03-27)

agc: https://github.com/cschin/agc.git revision: 453c0afdc54b4aa00fa8e97a63f196931fdb81c4 (git repo forked from https://github.com/

refresh-bio/agc)

PGR-TK: revision: 75fa20b41592941c9e6eef3f914d97788ee06b86 and after

Code repositora:

source: https://github.com/GeneDx/pgr-tk

API document: https://genedx.github.io/pgr-tk/

pre-built binaries for amd64 Ubuntu 20.04 linux distribution

https://github.com/GeneDx/pgr-tk/releases/tag/v0.4.0

The licenses of the code / usage limitation: https://github.com/GeneDx/pgr-tk/blob/main/LICENSE
Additional examples can be found at https://github.com/GeneDX/pgr-tk and https://github.com/Sema4-Research/pgr-tk-notebooks

For manuscripts utilizing custom algorithms or software that are central to the research but not yet described in published literature, software must be made available to editors and reviewers. We strongly encourage code deposition in a community repository (e.g. GitHub). See the Nature Research guidelines for submitting code & software for further information.

Data

Policy information about availability of data

All manuscripts must include a data availability statement. This statement should provide the following information, where applicable:

- Accession codes, unique identifiers, or web links for publicly available datasets
- A list of figures that have associated raw data
- A description of any restrictions on data availability

The initial assembly contigs are downloaded from AWS S3, the full GenBank Accessions are in https://github.com/GeneDx/pgr-tk-notebooks/blob/main/pgr-tk-sequence-source/genbank_accessions.txt. The URLs that we download are in https://github.com/GeneDx/pgr-tk-notebooks/blob/main/pgr-tk-sequence-source/Year1_assemblies_v2_genbank.index, we list them as well:

s3://human-pangenomics/working/HPRC/HG00438/assemblies/year1_f1_assembly_v2_genbank/HG00438.paternal.f1_assembly_v2_genbank.fa.gz
s3://human-pangenomics/working/HPRC/HG00438/assemblies/year1_f1_assembly_v2_genbank/HG00438.maternal.f1_assembly_v2_genbank.fa.gz
s3://human-pangenomics/working/HPRC/HG00621/assemblies/year1_f1_assembly_v2_genbank/HG00621.paternal.f1_assembly_v2_genbank.fa.gz
s3://human-pangenomics/working/HPRC/HG00621/assemblies/year1_f1_assembly_v2_genbank/HG00621.maternal.f1_assembly_v2_genbank.fa.gz
s3://human-pangenomics/working/HPRC/HG00673/assemblies/year1_f1_assembly_v2_genbank/HG00673.paternal.f1_assembly_v2_genbank.fa.gz
s3://human-pangenomics/working/HPRC/HG00673/assemblies/year1_f1_assembly_v2_genbank/HG00673.maternal.f1_assembly_v2_genbank.fa.gz
s3://human-pangenomics/working/HPRC/HG00735/assemblies/year1_f1_assembly_v2_genbank/HG00735.paternal.f1_assembly_v2_genbank.fa.gz
s3://human-pangenomics/working/HPRC/HG00735/assemblies/year1_f1_assembly_v2_genbank/HG00735.maternal.f1_assembly_v2_genbank.fa.gz
s3://human-pangenomics/working/HPRC/HG00741/assemblies/year1_f1_assembly_v2_genbank/HG00741.paternal.f1_assembly_v2_genbank.fa.gz
s3://human-pangenomics/working/HPRC/HG00741/assemblies/year1_f1_assembly_v2_genbank/HG00741.maternal.f1_assembly_v2_genbank.fa.gz
s3://human-pangenomics/working/HPRC/HG01071/assemblies/year1_f1_assembly_v2_genbank/HG01071.paternal.f1_assembly_v2_genbank.fa.gz
s3://human-pangenomics/working/HPRC/HG01071/assemblies/year1_f1_assembly_v2_genbank/HG01071.maternal.f1_assembly_v2_genbank.fa.gz
s3://human-pangenomics/working/HPRC/HG01106/assemblies/year1_f1_assembly_v2_genbank/HG01106.paternal.f1_assembly_v2_genbank.fa.gz
s3://human-pangenomics/working/HPRC/HG01106/assemblies/year1_f1_assembly_v2_genbank/HG01106.maternal.f1_assembly_v2_genbank.fa.gz
s3://human-pangenomics/working/HPRC/HG01123/assemblies/year1_f1_assembly_v2_genbank/HG01123.paternal.f1_assembly_v2_genbank.fa.gz
s3://human-pangenomics/working/HPRC/HG01123/assemblies/year1_f1_assembly_v2_genbank/HG01123.maternal.f1_assembly_v2_genbank.fa.gz
s3://human-pangenomics/working/HPRC/HG01175/assemblies/year1_f1_assembly_v2_genbank/HG01175.paternal.f1_assembly_v2_genbank.fa.gz
s3://human-pangenomics/working/HPRC/HG01175/assemblies/year1_f1_assembly_v2_genbank/HG01175.maternal.f1_assembly_v2_genbank.fa.gz
s3://human-pangenomics/working/HPRC/HG01258/assemblies/year1_f1_assembly_v2_genbank/HG01258.paternal.f1_assembly_v2_genbank.fa.gz
s3://human-pangenomics/working/HPRC/HG01258/assemblies/year1_f1_assembly_v2_genbank/HG01258.maternal.f1_assembly_v2_genbank.fa.gz
s3://human-pangenomics/working/HPRC/HG01358/assemblies/year1_f1_assembly_v2_genbank/HG01358.paternal.f1_assembly_v2_genbank.fa.gz
s3://human-pangenomics/working/HPRC/HG01358/assemblies/year1_f1_assembly_v2_genbank/HG01358.maternal.f1_assembly_v2_genbank.fa.gz
s3://human-pangenomics/working/HPRC/HG01361/assemblies/year1_f1_assembly_v2_genbank/HG01361.paternal.f1_assembly_v2_genbank.fa.gz
s3://human-pangenomics/working/HPRC/HG01361/assemblies/year1_f1_assembly_v2_genbank/HG01361.maternal.f1_assembly_v2_genbank.fa.gz
s3://human-pangenomics/working/HPRC/HG01891/assemblies/year1_f1_assembly_v2_genbank/HG01891.paternal.f1_assembly_v2_genbank.fa.gz
s3://human-pangenomics/working/HPRC/HG01891/assemblies/year1_f1_assembly_v2_genbank/HG01891.maternal.f1_assembly_v2_genbank.fa.gz
s3://human-pangenomics/working/HPRC/HG01928/assemblies/year1_f1_assembly_v2_genbank/HG01928.paternal.f1_assembly_v2_genbank.fa.gz
s3://human-pangenomics/working/HPRC/HG01928/assemblies/year1_f1_assembly_v2_genbank/HG01928.maternal.f1_assembly_v2_genbank.fa.gz
s3://human-pangenomics/working/HPRC/HG01952/assemblies/year1_f1_assembly_v2_genbank/HG01952.paternal.f1_assembly_v2_genbank.fa.gz
s3://human-pangenomics/working/HPRC/HG01952/assemblies/year1_f1_assembly_v2_genbank/HG01952.maternal.f1_assembly_v2_genbank.fa.gz
s3://human-pangenomics/working/HPRC/HG01978/assemblies/year1_f1_assembly_v2_genbank/HG01978.paternal.f1_assembly_v2_genbank.fa.gz
s3://human-pangenomics/working/HPRC/HG01978/assemblies/year1_f1_assembly_v2_genbank/HG01978.maternal.f1_assembly_v2_genbank.fa.gz
s3://human-pangenomics/working/HPRC/HG02148/assemblies/year1_f1_assembly_v2_genbank/HG02148.paternal.f1_assembly_v2_genbank.fa.gz
s3://human-pangenomics/working/HPRC/HG02148/assemblies/year1_f1_assembly_v2_genbank/HG02148.maternal.f1_assembly_v2_genbank.fa.gz
s3://human-pangenomics/working/HPRC/HG02257/assemblies/year1_f1_assembly_v2_genbank/HG02257.paternal.f1_assembly_v2_genbank.fa.gz
s3://human-pangenomics/working/HPRC/HG02257/assemblies/year1_f1_assembly_v2_genbank/HG02257.maternal.f1_assembly_v2_genbank.fa.gz
s3://human-pangenomics/working/HPRC/HG02486/assemblies/year1_f1_assembly_v2_genbank/HG02486.paternal.f1_assembly_v2_genbank.fa.gz
s3://human-pangenomics/working/HPRC/HG02486/assemblies/year1_f1_assembly_v2_genbank/HG02486.maternal.f1_assembly_v2_genbank.fa.gz
s3://human-pangenomics/working/HPRC/HG02559/assemblies/year1_f1_assembly_v2_genbank/HG02559.paternal.f1_assembly_v2_genbank.fa.gz
s3://human-pangenomics/working/HPRC/HG02559/assemblies/year1_f1_assembly_v2_genbank/HG02559.maternal.f1_assembly_v2_genbank.fa.gz
s3://human-pangenomics/working/HPRC/HG02572/assemblies/year1_f1_assembly_v2_genbank/HG02572.paternal.f1_assembly_v2_genbank.fa.gz

s3://human-pangenomics/working/HPRC/HG02572/assemblies/year1_f1_assembly_v2_genbank/HG02572.maternal.f1_assembly_v2_genbank.fa.gz
s3://human-pangenomics/working/HPRC/HG02622/assemblies/year1_f1_assembly_v2_genbank/HG02622.paternal.f1_assembly_v2_genbank.fa.gz
s3://human-pangenomics/working/HPRC/HG02622/assemblies/year1_f1_assembly_v2_genbank/HG02622.maternal.f1_assembly_v2_genbank.fa.gz
s3://human-pangenomics/working/HPRC/HG02630/assemblies/year1_f1_assembly_v2_genbank/HG02630.paternal.f1_assembly_v2_genbank.fa.gz
s3://human-pangenomics/working/HPRC/HG02630/assemblies/year1_f1_assembly_v2_genbank/HG02630.maternal.f1_assembly_v2_genbank.fa.gz
s3://human-pangenomics/working/HPRC/HG02717/assemblies/year1_f1_assembly_v2_genbank/HG02717.paternal.f1_assembly_v2_genbank.fa.gz
s3://human-pangenomics/working/HPRC/HG02717/assemblies/year1_f1_assembly_v2_genbank/HG02717.maternal.f1_assembly_v2_genbank.fa.gz
s3://human-pangenomics/working/HPRC/HG02886/assemblies/year1_f1_assembly_v2_genbank/HG02886.paternal.f1_assembly_v2_genbank.fa.gz
s3://human-pangenomics/working/HPRC/HG02886/assemblies/year1_f1_assembly_v2_genbank/HG02886.maternal.f1_assembly_v2_genbank.fa.gz
s3://human-pangenomics/working/HPRC/HG03453/assemblies/year1_f1_assembly_v2_genbank/HG03453.paternal.f1_assembly_v2_genbank.fa.gz
s3://human-pangenomics/working/HPRC/HG03453/assemblies/year1_f1_assembly_v2_genbank/HG03453.maternal.f1_assembly_v2_genbank.fa.gz
s3://human-pangenomics/working/HPRC/HG03516/assemblies/year1_f1_assembly_v2_genbank/HG03516.paternal.f1_assembly_v2_genbank.fa.gz
s3://human-pangenomics/working/HPRC/HG03516/assemblies/year1_f1_assembly_v2_genbank/HG03516.maternal.f1_assembly_v2_genbank.fa.gz
s3://human-pangenomics/working/HPRC/HG03540/assemblies/year1_f1_assembly_v2_genbank/HG03540.paternal.f1_assembly_v2_genbank.fa.gz
s3://human-pangenomics/working/HPRC/HG03540/assemblies/year1_f1_assembly_v2_genbank/HG03540.maternal.f1_assembly_v2_genbank.fa.gz
s3://human-pangenomics/working/HPRC/HG03579/assemblies/year1_f1_assembly_v2_genbank/HG03579.paternal.f1_assembly_v2_genbank.fa.gz
s3://human-pangenomics/working/HPRC/HG03579/assemblies/year1_f1_assembly_v2_genbank/HG03579.maternal.f1_assembly_v2_genbank.fa.gz
s3://human-pangenomics/working/HPRC_PLUS/HG002/assemblies/year1_f1_assembly_v2_genbank/HG002.paternal.f1_assembly_v2_genbank.fa.gz
s3://human-pangenomics/working/HPRC_PLUS/HG002/assemblies/year1_f1_assembly_v2_genbank/HG002.maternal.f1_assembly_v2_genbank.fa.gz
s3://human-pangenomics/working/HPRC_PLUS/HG005/assemblies/year1_f1_assembly_v2_genbank/HG005.paternal.f1_assembly_v2_genbank.fa.gz
s3://human-pangenomics/working/HPRC_PLUS/HG005/assemblies/year1_f1_assembly_v2_genbank/HG005.maternal.f1_assembly_v2_genbank.fa.gz
s3://human-pangenomics/working/HPRC_PLUS/HG00733/assemblies/year1_f1_assembly_v2_genbank/HG00733.paternal.f1_assembly_v2_genbank.fa.gz
s3://human-pangenomics/working/HPRC_PLUS/HG00733/assemblies/year1_f1_assembly_v2_genbank/HG00733.maternal.f1_assembly_v2_genbank.fa.gz
s3://human-pangenomics/working/HPRC_PLUS/HG01109/assemblies/year1_f1_assembly_v2_genbank/HG01109.paternal.f1_assembly_v2_genbank.fa.gz
s3://human-pangenomics/working/HPRC_PLUS/HG01109/assemblies/year1_f1_assembly_v2_genbank/HG01109.maternal.f1_assembly_v2_genbank.fa.gz
s3://human-pangenomics/working/HPRC_PLUS/HG01243/assemblies/year1_f1_assembly_v2_genbank/HG01243.paternal.f1_assembly_v2_genbank.fa.gz
s3://human-pangenomics/working/HPRC_PLUS/HG01243/assemblies/year1_f1_assembly_v2_genbank/HG01243.maternal.f1_assembly_v2_genbank.fa.gz
s3://human-pangenomics/working/HPRC_PLUS/HG02055/assemblies/year1_f1_assembly_v2_genbank/HG02055.paternal.f1_assembly_v2_genbank.fa.gz
s3://human-pangenomics/working/HPRC_PLUS/HG02055/assemblies/year1_f1_assembly_v2_genbank/HG02055.maternal.f1_assembly_v2_genbank.fa.gz
s3://human-pangenomics/working/HPRC_PLUS/HG02080/assemblies/year1_f1_assembly_v2_genbank/HG02080.paternal.f1_assembly_v2_genbank.fa.gz
s3://human-pangenomics/working/HPRC_PLUS/HG02080/assemblies/year1_f1_assembly_v2_genbank/HG02080.maternal.f1_assembly_v2_genbank.fa.gz
s3://human-pangenomics/working/HPRC_PLUS/HG02109/assemblies/year1_f1_assembly_v2_genbank/HG02109.paternal.f1_assembly_v2_genbank.fa.gz
s3://human-pangenomics/working/HPRC_PLUS/HG02109/assemblies/year1_f1_assembly_v2_genbank/HG02109.maternal.f1_assembly_v2_genbank.fa.gz
s3://human-pangenomics/working/HPRC_PLUS/HG02145/assemblies/year1_f1_assembly_v2_genbank/HG02145.paternal.f1_assembly_v2_genbank.fa.gz
s3://human-pangenomics/working/HPRC_PLUS/HG02145/assemblies/year1_f1_assembly_v2_genbank/HG02145.maternal.f1_assembly_v2_genbank.fa.gz
s3://human-pangenomics/working/HPRC_PLUS/HG02723/assemblies/year1_f1_assembly_v2_genbank/HG02723.paternal.f1_assembly_v2_genbank.fa.gz
s3://human-pangenomics/working/HPRC_PLUS/HG02723/assemblies/year1_f1_assembly_v2_genbank/HG02723.maternal.f1_assembly_v2_genbank.fa.gz
s3://human-pangenomics/working/HPRC_PLUS/HG02818/assemblies/year1_f1_assembly_v2_genbank/HG02818.paternal.f1_assembly_v2_genbank.fa.gz
s3://human-pangenomics/working/HPRC_PLUS/HG02818/assemblies/year1_f1_assembly_v2_genbank/HG02818.maternal.f1_assembly_v2_genbank.fa.gz
s3://human-pangenomics/working/HPRC_PLUS/HG03098/assemblies/year1_f1_assembly_v2_genbank/HG03098.paternal.f1_assembly_v2_genbank.fa.gz
s3://human-pangenomics/working/HPRC_PLUS/HG03098/assemblies/year1_f1_assembly_v2_genbank/HG03098.maternal.f1_assembly_v2_genbank.fa.gz
s3://human-pangenomics/working/HPRC_PLUS/HG03486/assemblies/year1_f1_assembly_v2_genbank/HG03486.paternal.f1_assembly_v2_genbank.fa.gz
s3://human-pangenomics/working/HPRC_PLUS/HG03486/assemblies/year1_f1_assembly_v2_genbank/HG03486.maternal.f1_assembly_v2_genbank.fa.gz
s3://human-pangenomics/working/HPRC_PLUS/HG03492/assemblies/year1_f1_assembly_v2_genbank/HG03492.paternal.f1_assembly_v2_genbank.fa.gz
s3://human-pangenomics/working/HPRC_PLUS/HG03492/assemblies/year1_f1_assembly_v2_genbank/HG03492.maternal.f1_assembly_v2_genbank.fa.gz
s3://human-pangenomics/working/HPRC_PLUS/NA18906/assemblies/year1_f1_assembly_v2_genbank/NA18906.paternal.f1_assembly_v2_genbank.fa.gz
s3://human-pangenomics/working/HPRC_PLUS/NA18906/assemblies/year1_f1_assembly_v2_genbank/NA18906.maternal.f1_assembly_v2_genbank.fa.gz
s3://human-pangenomics/working/HPRC_PLUS/NA19240/assemblies/year1_f1_assembly_v2_genbank/NA19240.paternal.f1_assembly_v2_genbank.fa.gz
s3://human-pangenomics/working/HPRC_PLUS/NA19240/assemblies/year1_f1_assembly_v2_genbank/NA19240.maternal.f1_assembly_v2_genbank.fa.gz
s3://human-pangenomics/working/HPRC_PLUS/NA20129/assemblies/year1_f1_assembly_v2_genbank/NA20129.paternal.f1_assembly_v2_genbank.fa.gz
s3://human-pangenomics/working/HPRC_PLUS/NA20129/assemblies/year1_f1_assembly_v2_genbank/NA20129.maternal.f1_assembly_v2_genbank.fa.gz
s3://human-pangenomics/working/HPRC_PLUS/NA21309/assemblies/year1_f1_assembly_v2_genbank/NA21309.paternal.f1_assembly_v2_genbank.fa.gz
s3://human-pangenomics/working/HPRC_PLUS/NA21309/assemblies/year1_f1_assembly_v2_genbank/NA21309.maternal.f1_assembly_v2_genbank.fa.gz
s3://human-pangenomics/working/HPRC_PLUS/CHM13/assemblies/chm13.draft_v1.1.fasta.gz
s3://human-pangenomics/working/HPRC_PLUS/GRCh38/assemblies/GCA_000001405.15_GRCh38_no_alt_analysis_set.fna.gz

The HPRC year one release sequence and pre-built index: https://giab-data.s3.amazonaws.com/PGR-TK-Files/pgr-tk-HGRP-y1-evaluation-set-v0.tar

All GFA files, fetched sequences from HPRC year one release of the 385 CMRG: https://giab-data.s3.amazonaws.com/PGR-TK-Files/CMRG_output_dir_v0.3.3.tar
Example Notebooks using PGR-TK including code making most of (the source of) the plots in this manuscript: https://github.com/Sema4-Research/pgr-tk-notebooks

Information about a docker image with prebuilt PGR-TK library and Jupyter Lab Server and Usage: https://github.com/Sema4-Research/pgr-tk/blob/main/pgr-tk-workstation/Readme.md

Field-specific reporting

Please select the one below that is the best fit for your research. If you are not sure, read the appropriate sections before making your selection.

☒ Life sciences ☐ Behavioural & social sciences ☐ Ecological, evolutionary & environmental sciences

Life sciences study design

All studies must disclose on these points even when the disclosure is negative.

Sample size	Not applicable, our work is on bioinformatics methods not control-case study. The amount data we used are just limited by the amount of genome generated by HPRC or other agencies and computational resource available to us.
Data exclusions	not applicable, see above.
Replication	Not applicable, see above. (The code is totally deterministic, although some code / algorithm improvement may changes the results. Once the code revision is fix, with the same input data, we will get the same output results.)
Randomization	Not applicable, Our results does not dependent objective statistic analysis.
Blinding	Not applicable, Our results does not dependent objective statistic analysis.

Reporting for specific materials, systems and methods

We require information from authors about some types of materials, experimental systems and methods used in many studies. Here, indicate whether each material, system or method listed is relevant to your study. If you are not sure if a list item applies to your research, read the appropriate section before selecting a response.

Materials & experimental systems

n/a	Involved in the study	
☒	☐	Antibodies
☒	☐	Eukaryotic cell lines
☒	☐	Palaeontology and archaeology
☒	☐	Animals and other organisms
☒	☐	Human research participants
☒	☐	Clinical data
☒	☐	Dual use research of concern

Methods

n/a	Involved in the study	
☒	☐	ChIP-seq
☒	☐	Flow cytometry
☒	☐	MRI-based neuroimaging
